# Transorbital endoscopic approaches to the skull base: a systematic literature review and anatomical description

**DOI:** 10.1007/s10143-020-01470-5

**Published:** 2021-01-22

**Authors:** Alperen Vural, Andrea Luigi Camillo Carobbio, Marco Ferrari, Vittorio Rampinelli, Alberto Schreiber, Davide Mattavelli, Francesco Doglietto, Barbara Buffoli, Luigi Fabrizio Rodella, Stefano Taboni, Michele Tomasoni, Tommaso Gualtieri, Alberto Deganello, Lena Hirtler, Piero Nicolai

**Affiliations:** 1 Department of Otorhinolaryngology, Erciyes University Faculty of Medicine, 38039 Kayseri, Turkey; 2 Unit of Otorhinolaryngology – Head and Neck Surgery, Department of Medical and Surgical Specialties, Radiological Sciences, and Public Health, University of Brescia, Piazzale Spedali Civili 1, 25123 Brescia, Italy; 3 IRCCS Ospedale Policlinico San Martino, Genoa, Italy; 4 Department of Surgical Sciences and Integrated Diagnostics (DISC), University of Genoa, Genoa, Italy; 5 Section of Otorhinolaryngology – Head and Neck Surgery, Department of Neurosciences, University of Padua, Padua, Italy; 6 Unit of Neurosurgery, Department of Medical and Surgical Specialties, Radiological Sciences, and Public Health, University of Brescia, Brescia, Italy; 7 Section of Anatomy and Physiopathology, Department of Clinical and Experimental Sciences, University of Brescia, Brescia, Italy; 8 Division of Anatomy, Center for Anatomy and Cell Biology, Medical University of Vienna, Vienna, Austria

**Keywords:** Endoscopy, Neuroendoscopy, Transorbital, Orbit, Skull base

## Abstract

Transorbital endoscopic approaches are increasing in popularity as they provide corridors to reach various areas of the ventral skull base through the orbit. They can be used either alone or in combination with different approaches when dealing with the pathologies of the skull base. The objective of the current study is to evaluate the surgical anatomy of transorbital endoscopic approaches by cadaver dissections as well as providing objective clinical data on their actual employment and morbidity through a systematic review of the current literature. Four cadaveric specimens were dissected, and step-by-step dissection of each endoscopic transorbital approach was performed to identify the main anatomic landmarks and corridors. A systematic review with pooled analysis of the current literature from January 2000 to April 2020 was performed and the related studies were analyzed. Main anatomical landmarks are presented based on the anatomical study and systematic review of the literature. With emphasis on the specific transorbital approach used, indications, surgical technique, and complications are reviewed through the systematic review of 42 studies (19 in vivo and 23 anatomical dissections) including 193 patients. In conclusion, transorbital endoscopic approaches are promising and appear as feasible techniques for the surgical treatment of skull base lesions. Surgical anatomy of transorbital endoscopic approaches can be mastered through knowledge of a number of anatomical landmarks. Based on data available in the literature, transorbital endoscopic approaches represent an important complementary that should be included in the armamentarium of a skull base team.

**Supplementary Information:**

The online version contains supplementary material available at 10.1007/s10143-020-01470-5.

## Introduction

Surgical approaches to the skull base (SB) significantly evolved over the last decades. Various meticulous anatomical studies have improved the understanding of SB anatomy from the endoscopic perspective, and transnasal endoscopic surgery has become the preferred approach for most pathologies of the median anterior SB and is being widely employed for a large number of lesions of the middle, posterior, and/or non-midline SB [42, 45, 49, 54, 62]. According to the contemporary literature, some tumors that were previously thought accessible only through open approaches are now being resected with a range of evolving novel techniques that exploit narrow anatomical corridors such as the sinonasal tract and orbit [47, 63].

Transnasal routes can be modified and combined depending upon the extent of the pathology, yet with the anatomical constraints posed by the course of relevant neurovascular structures [30, 62]. Although modifiable and extremely versatile, transnasal endoscopic approaches might provide inadequate access to lesions with far lateral extension. In these circumstances, the orbit appears a reliable portal to overcome this limit [2]. Transorbital endoscopic approaches (TEA) have been surmised to provide a direct route to the lateral portion of the SB. Consequently, they have been adopted with increasing frequency to resect SB lesions over the last decade [13, 15, 19, 25, 47, 48, 55, 57].

While initially limited to the pathologies of the orbit, TEAs are now used either alone or in combination with transnasal approaches, allowing to resect a wide range of pathologies of the SB while avoiding more extended and potentially disfiguring transfacial/transcranial techniques [7]. The term “transorbital neuroendoscopic surgery” (TONES) describes a group of endoscopic surgical corridors that may be indicated for several lesions affecting the anterior and middle cranial fossae. Understanding the surgical anatomy of TONES requires a certain eclecticism, as it covers areas that are usually approached by different physicians through other routes, and needs anatomical landmarks to be identified from the endoscopic perspective. This is rewarded with limited morbidity, neither visible scars nor external craniotomies, and minimal brain retraction [47]. Consequently, potential damage to adjacent neurovascular structures is held to a minimum, patient recovery is rapid, and hospitalization short [3, 6, 47, 57]. On the other hand, the enthusiasm raised by TEAs, which is witnessed by an increasing number of publications on this interesting topic, deserves to be weighted based on their genuine clinical indications and morbidity. This need contrasts with the fact that data on TEAs are heterogenous and fragmented throughout a number of single-institution publications.

The aim of the current study is to summarize the surgical anatomy of TEAs while providing objective clinical data on their actual employment and morbidity through a systematic review of the current literature.

## Materials and methods

### Anatomical study

Anatomical dissections were performed at the Laboratory of Endoscopic Anatomy of the University of Brescia (Brescia, Italy) and Division of Anatomy of the Medical University of Vienna (Vienna, Austria). Four fresh frozen cadaver heads were used. The specimens originated from voluntary body donations to the Division of Anatomy of the Medical University of Vienna (Vienna, Austria) (*n* = 3) or were provided by Medcure® (Portland, USA). Approval for the study by the local ethics commission was obtained (EC Nr. 1277/2016). Specimens were positioned supine, pinned, and fixed in a Mayfield head holder. Dissections were initiated with an external incision then continued endoscopically, following the surgical techniques described by Moe et al [47]. Endoscopic dissections were performed using a rigid 4-mm-diameter endoscope, 14 cm in length, with 0°, 45°, and 70° rod lenses. Images and videos were captured using a 4K digital video system (Olympus®, Japan). A high-speed drill was used for bone removal.

### Review protocol

The study protocol was designed in accordance with the Preferred Reporting Items for Systematic Reviews and Meta-Analysis (PRISMA) statement. The database search included Pubmed (Medline) and Scopus. Key words searched were ‘Transorbital’ and ‘Endoscopic Orbital’. Only English-language articles were included. Articles were screened and evaluated for eligibility excluding (1) case reports without anatomical studies; (2) transorbital procedures not including an endoscopic approach; (3) studies related to solely orbital pathologies; (4) letters to the editors, commentaries, reviews without cases or anatomical studies; and (5) other unrelated studies. The selected studies were included in the qualitative synthesis. The literature search was performed in April 2020 including only the studies published after 2000. Publications were reviewed based on title and abstract information to eliminate duplicate and irrelevant studies.

## RESULTS

### Literature review

**Fig. 1:**
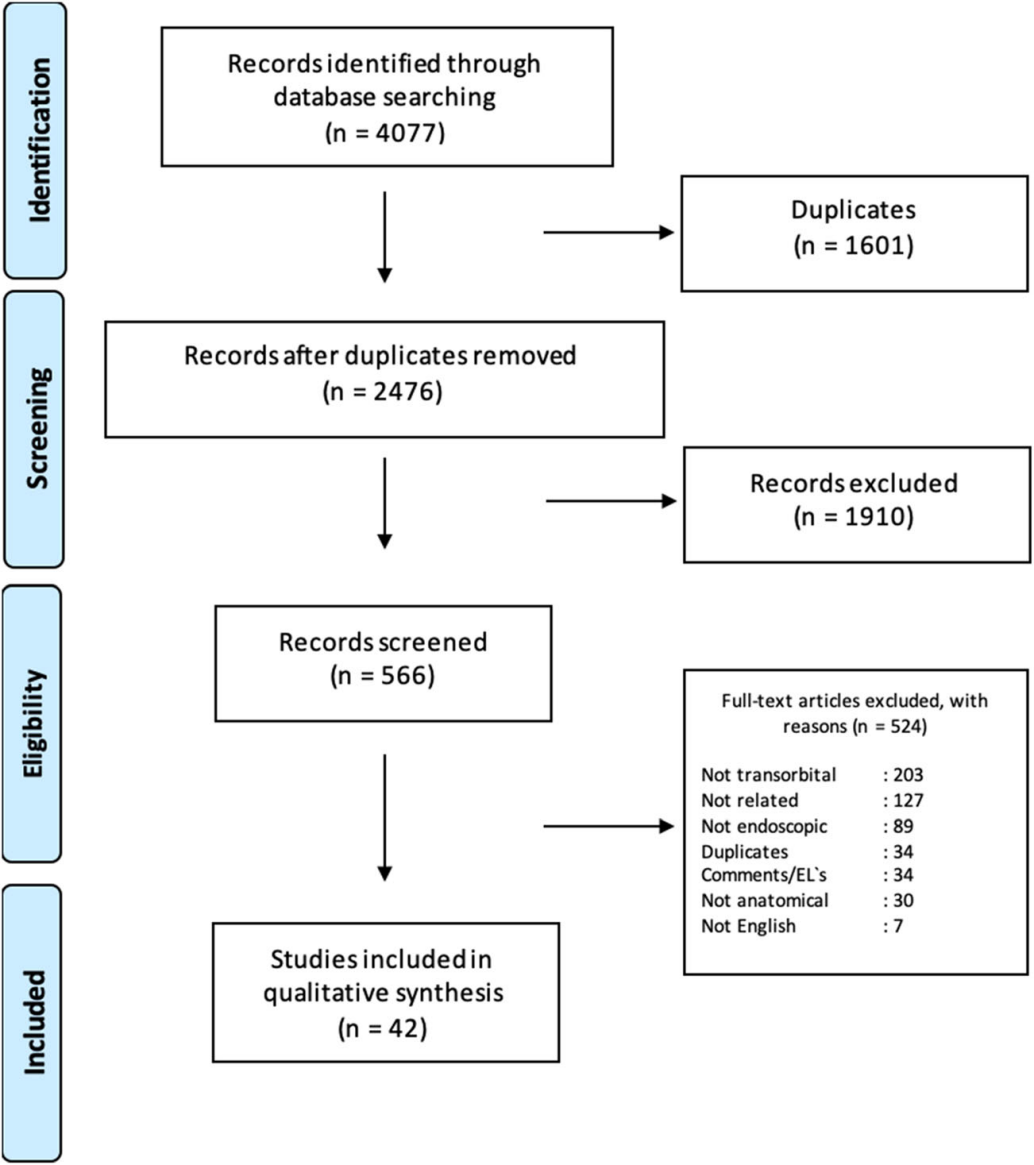
Diagram showing selection process based on Preferred Reporting Items for Systematic Reviews and Meta-Analyses (PRISMA) for the studies related to endoscopic transorbital approaches

**Fig. 2:**
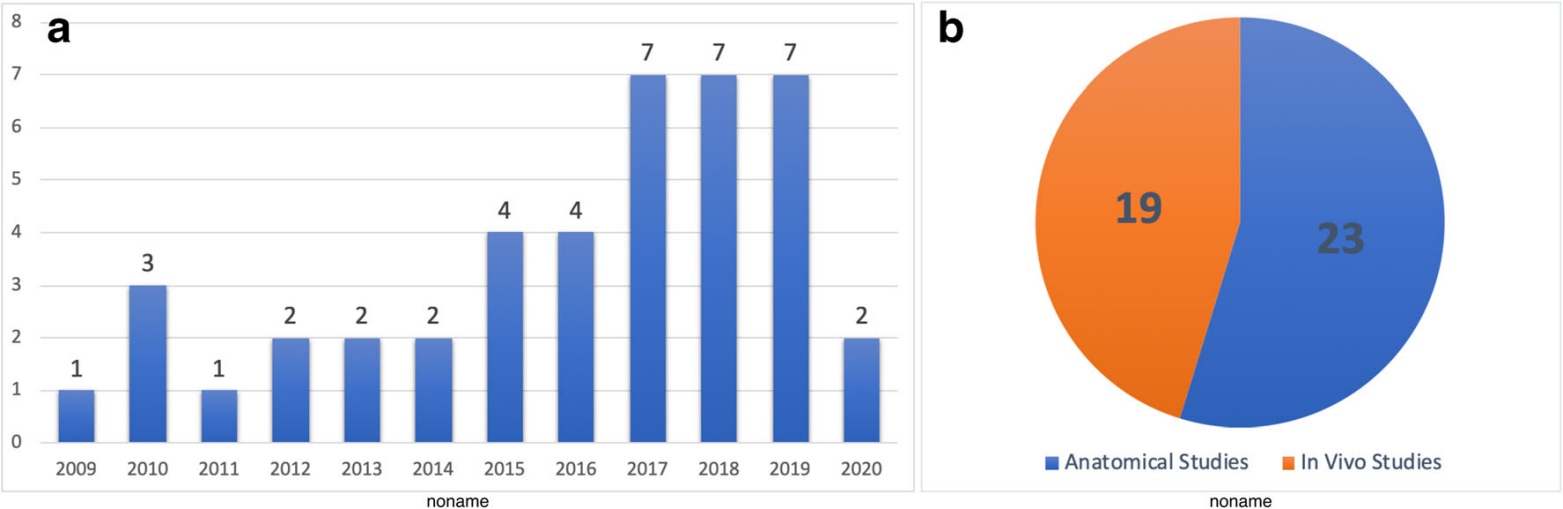
**A** Chart graph showing the distribution of the number of the articles published throughout the years. **B** Pie graph showing the distribution of the papers according to study type

The literature search with the keyword “Transorbital” revealed 435 records in Pubmed and 509 records in Scopus databases. Of those, 393 articles were duplicates. The search with the keyword “Endoscopic orbital” revealed 1502 records in Pubmed and 1631 records in Scopus of which 1208 were duplicates. After applying the exclusion criteria, a total of 42 studies were included in qualitative synthesis. Figure 1 presents the PRISMA flowchart summarizing identification, screening, eligibility, and inclusion criteria. An increasing trend of publications over the last years was observed (Fig. 2). Table 1 presents the studies including living patients (or both case series/report and a cadaver study), whereas Table 2 summarizes purely anatomical studies.

**Table 1:** Studies which include cases in vivo

Author	Year	Origin	Study type	Anatomical/surgical	N° of patients /specimen	Portals (n° of patients)	Approach	Craniectomy	Reconstruction	Target area	Surgical landmarks
Park [53]	2020	South Korea	CSt	Sx	24 patients (11 TO)	TO (vs minipterional approach) (11)	SLC	Drilling of GSW	Fascia lata or AlloDerm, with TachoSil, abdominal fat	Sphenoid wing	SOF, OC, IOF, MLA, GSW, MOB, TM
Gerges [27]	2019	USA	LI + Case	An + Sx	4 cadaver heads + 1 patient	TO (1)	Inferior eyelid	None	None	ITF, PS	IOF, GSW, TM, ZR, LPM
De Rosa [16]	2019	Spain/Italy	LI + Case	An + Sx	3 cadaver heads + 1 patient	TO + (endoscopic extraorbital) (1)	SLC + Lateral canthotomy (for extraorbital corridor)	Drilling of GSW and LSW	Tisseel, Fat graft	FR, FO, SOF	SOF, IOF + TM, pterion (for extraorbital corridor)
Lee [34]	2019	South Korea	CSt	Sx	21 patients (9 TO)	TO (7), TO + TN (2)	SLC	Removal of LOW and vertical crest	2 layers autologous fascia or alloderm + suturing of the dura	MCF, CS	SOF, MOB, V1, V2
Golbin [28]	2019	Russia	CSt	Sx	12 patients (6 biopsies, 6 resections)	TO (9), TO + TN (3)	SLC (8), retrocaruncular (2), lateral retrocanthal (1), upper medial (1)	Not mentioned	Fat graft, fascia lata (3 layer)	N/A	SOF, IOF, GSW, FR, FO, FS (for SL approach) - ALC, lacrimal ethmoid suture, AEA, PEA, OC (for RC)
Lubbe [41]	2019	South Africa	LI + case	Sx + An	1 patient, 1 cadaver head	TO + TN (1)	Contralateral PC	N/A	Abdominal fat, DuraGen, NSF	Lateral recess of the sphenoid sinus	AEA, ST
Kong [31]	2018	South Korea	CSt	Sx	18 patients	TO (16), TO + TN (2)	SLC	N/A	Double layer fascia lata or alloderm and fat if needed	N/A	N/A
Jeon [29]	2018	South Korea	CSt	Sx	9 patients	TO (8), TO + TN (1), Suboccipital craniotomy (1)	SLC	Removal of LOW, vertical crest of IOF, and GSW. Dural incision was made to reach the temporal lobe. İn lesions limited to the MC, an interdural approach reaching the lateral border of the CS	TachoSil, double layer autologous fascia or AlloDerm + Medpor + Miniplate	MC, TL	SOF, GSW, IOF, MOB
Dallan [15]	2018	Italy	CSt	Sx	14 patients	TO (10), TO + TN (4)	SLC	In 2 patients with intradural extension through GSW	Multilayer, fascia lata, intradural fat	N/A	IOF, SOF, GSW, TM, MCF dura
Lubbe [40]	2017	South Africa	CSt	Sx	7 patients	TO + TN (7)	LRC	Drilling of GSW in addition to removal of LOW (craniectomy in 1 pt)	Underlay DuraGen graft	Orbit, Sphenoid	TM, LOW, GSW
Chen [8]	2015	USA	Case	Sx	2 patients	TO (2)	SLC (1), SLC extended laterally from lateral canthus (1)	Drilling of GSW between SOF and IOF (in one case additional LOW removal)	Free local tissue graft, dural sealant	Hippocampus, amygdala, entorhinal cortex	SOF, IOF
Dallan [12]	2015	Italy	LI + CSt	An + Sx	5 cadaver heads + 4 cases	TO + TN (4)	SLC + inferior eyelid crease	By removal of GSW and extended inferiorly if MCF exposure is needed	ITT + Fat	MCF, CS, V2, V3, ON, ICA, temporal lobe	IOF, SOF, TM, AEA, PEA
Lyson [43]	2014	Poland	Case	Sx	1 patient	TO	Lateral orbitotomy	N/A	Tachosil (for the orbit)	Orbit	TM
Raza [58]	2013	USA	CSt	Sx	6 patients	TO + TN (4)	PC	Minicraniectomy along the superomedial aspect of the orbit	Fascia lata	Planum sphenoidale (2), ACF (2), cribriform plate, medial orbital roof	AEA, PEA, FES
Koppe [32]	2013	France	CSt	Sx	10 patients	TO	SLC	Supraorbital drilling	Dural suture, fat graft	Sella	N/A
Lim [36]	2012	USA	CSt	Sx	13 patients	TO	PC (4), SLC (9)	N/A	N/A	frontal sinus, orbit, ACF	N/A
Balakrishnan [3]	2011	USA	CSt	Sx	107 patients	TO	LRC (50), LE (65), PC (55), SLC (17)	N/A	N/A	N/A	N/A
Moe [48]	2011	USA	LI + CSt	An + Sx	5 cadaver heads + 10 patients	TO	SLC + PC (in each cadaver), SLC (5), PC (4), SLC + PC + PSA + LRC (1)	N/A	For supraorbital defects one-layer allograft, in interorbital defects, 2 layers of allograft + bioglue + Hadad flap (in some cases)	ACF	N/A
Moe [47]	2010	USA	LI + CSt	An + Sx	3 cadaver heads + 16 patients	TO	LRC (1), SLC (6), PC (7), PS (1), LRC + SLC + PC + PS (1)	Removal of GSW	N/A	OA, sella, ACF (for PC), ant temporal lobe, MCF (LRC), FR (PS), supraorbital ACF (SLC)	AEA, PEA

*ACF* anterior cranial fossa, *ACP* anterior clinoid process, *AEA* anterior ethmoidal artery, *ALC* anterior lacrimal crest, *An* anatomical, *CS* cavernous sinus, *CSt* clinical study, *EOA* endoscopic orbital approach, *FES* frontoethmoid suture, *FO* foramen ovale, *FR* foramen rotundum, *FS* foramen spinosum, *FZS* frontozygomatic suture, *GG* Gasserian ganglion, *GSW* greater sphenoidal wing, *ICA* internal carotid artery, *Inf* inferior, *IOF* inferior orbital fissure, *ITF* infratemporal fossa, *KT* Kawase triangle, *Lat* lateral, *LI* laboratory investigation, *LOW* lateral orbital wall, *LRC* lateral retrocanthal, *LSW* lesser sphenoidal wing, *MC* Meckel`s cave, *MCF* middle cranial fossa, *Med* medial, *MIS* middle incisural space, *MLA* meningolacrimal artery, *MMA* middle meningeal artery, *MOB* meningoorbital band, *MOW* medial orbital wall, *N/A* not applicable, *OA* orbital apex, *OC* optic canal, *ON* optic nerve, *PC* precaruncular, *PCF* posterior cranial fossa, *PEA* posterior ethmoidal artery, *PS* preseptal lower eyelid, *SF* supraorbital foramen, *SLC* superior eyelid crease, *SOF* superior orbital fissure, *Sp* sphenoid, *ST* superior turbinate, *Sup* superior, *Sx* surgical, *TL* temporal lobe, *TM* temporalis muscle, *TN* transnasal, *TO* transorbital, *VC* Vidian canal, *VN* Vidian nerve, *ZF* zygomaticotemporal foramen, *ZFB* zygomaticofacial bundle, *ZTB* zygomaticotemporal bundle

**Table 2:** Anatomical studies

Author	Year	Origin	Nr of specimens	Portals	Approach	Craniectomy	Reconstruction	Target area	Surgical landmarks
Saraceno [59]	2020	Italy	5 heads	TO	SLC and ILTEA	Drilling the GSW	N/A	MCF	SOF, IOF, TM
Bon-Jour Lin [38]	2019	China	5 heads	TO	Lateral canthotomy with cantholysis + preseptal lower eyelid	Removal of GSW, drilling between FR and FO	Titanium Mesh, Miniplates	FR, FO, PPF, ITF, MCF, MC, GG, LWCS	GSW, MOB, ION, SOF, TM, IOF
Laleva [33]	2019	Bulgaria	3 heads	TO	SLC extending through the zygoma	By removal of LOW and sphenoid ridge	N/A	Anteromedial: ACP, optic canal, ON, ICA; Posteromedial: LWCS; Posterior: MC, petrous apex; Inferior: ITF, pterygoid fossa	ZF, SF, TM, FZS, sphenoid ridge, MOB, ACP, LSW, SOF, GSW, FR, VC, FO
Bon-Jour Lin [37]	2019	China	4 heads	TO	SLC + Lateral canthotomy and cantholysis	Large bone drilling of the GSW, LOW, SOW to reach to ACF and MCF dura	N/A	MIS, tentorium, MC, interpeduncular cistern, prepontine cistern	MLA, MOB, SOF, OC, IOF, anterior clinoid, M1 of MCA
Noiphitak [52]	2018	USA	7 heads	TO + (endoscopic extraorbital corridor)	Extended incision from the lateral canthus towards lateral + canthotomy	Removal of LOW, drilling from IOF to SOF. Dural incision was made to reach the temporal lobe	N/A	MIS, tentorium, MC, interpeduncular cistern, prepontine cistern	MLA, MOB, SOF, OC, IOF, ACP, M1 of MCA
Noiphitak [51]	2018	USA	5 heads	TO	SLC	Removal of SOW, laterally from the SOF to FS, removal of LOW from SOF to TM and from SOF to IOF	None	ACF, MCF, ICA, ACA, Chiasm, MCA	SOF, IOF, AEA, PEA, OC, MOF
Noiphitak [50]	2018	USA	5 heads	TO + (endoscopic extraorbital corridor) + anterior transpetrosal	Extended incision from the lateral canthus towards canthotomy	N/A	N/A	Infratentorial region, PF, CN IV, V, VII, VIII, most anterosuperior, anteroinferior and posterosuperior accesible points of the brainstem	LOW, TM, GWS, Temporal dura, GSPN, LSPN, MMA, CNV1-3, Kawase triangle, IAC, tentorium cerebelli, CN IV
Di Somma [20]	2018	Italy	5 heads	TO + Supraorbital	SLC (+ eyebrow incision)	Initially performed through zygoma (temporal fossa) and continued though GSW	N/A	Parasellar and lateral MCF (i.e. Sylvian fissure), MCA, most inferior visible point of CS	IOF, SOF, GSW, LSW, MOB, ACP, ICA
Di Somma [21]	2018	Italy	5 heads	TO + Supraorbital	SLC	Initially performed through zygoma (temporal fossa) and continued though GSW	N/A	Petrous bone, Cerebellopontine angle space, MIS, Ventral brainstem space	SOF, TM, IOF, MMA, FS, FO, MOB, GSPN, pICA, GG, tentorium
Di Somma [19]	2017	Italy	10 heads	TO	SLC	4 types proposed: 1) lateral corridor to MCF, 2) lateral corridor to ACF, 3) combined lateral to MCF and ACF with LSW removal and 4) medial corridor to opticocarotid region	N/A	ACF, MCF	GSW, SOF, LSW, TM, IOF, MMA, MOB, MCA
Almeida [1]	2017	USA	4 heads	TO + (TN)	SLC	Via drilling the orbital roof and GSW. TM is the lateral limit for craniectomy.	2-layer temporal fascia graft	Sylvian fissure, MCA, AL surface of insula, ICA, crural and ambiens cistern	MLA, SOF, IOF, TM, ACP, MCA
Priddy [56]	2017	USA	9 heads	TO	SLC	Drilling of GSW and LSW	N/A	MC	SOF, MOB, LOW, GSW
Dallan [14]	2017	Italy	5 heads	TO	SLC	Drilling of GSW	N/A	CS	SOF, MLA, GSW, MOB
Di Somma [21]	2017	Italy	5 heads	TO + (TN)	SLC	Until optic chiasm by removal of ACP	N/A	ON, OC	SOF, OC, PEA, ICA
Ciporen [11]	2017	USA	3 heads	Transnasal (clival) compared with TN + TO	PC	Transnasal transclival	N/A	Posterior cerebral vessels (BA – proximal to its apex –, PCA, SCA, and AICA)	AEA, PEA, lamina papyracea
Ciporen [10]	2016	USA	8 heads	Transnasal (clival) compared with TN+TO	PC	Transnasal transclival	N/A	Cavernous ICA	AEA, PEA, lamina papyracea
Matsuo [44]	2016	USA	7 orbits	Translateral orbit	Translateral orbital wall approach (orbitozygomatic approach)	LOW, GSW osteotomy	N/A	Superior. and lateral surfaces of the orbit, OC, SOF, and CS (after drilling the GSW and ACP MCF, KT could be reached)	TM, LOW, GSW, SOF
Ferrari [25]	2016	Italy	7 heads	TO	Inferolateral orbital rim	Triangle between SOF and IOF exposing TM, ITF, and MCF	N/A	4 corridors: MC corridor (GG, SPS), carotid foramen (ET, ICA), petrous corridor (GSPN, EA), transdural MCF corridor (medial surface of TL, temporal horn of lateral ventricle, tentorium)	ZFB, ZTB, IOF, SOF, MOB, MLA, FO, V2, V3, MMA, FS, FR
Alqahtani [2]	2015	Italy	5 heads	TO + TN	Transpalpebral (transverse supratarsal skin incision)	By removal of superior orbital wall	Multilayer with synthetic graft, mucoperiosteal septal graft	ACF, MCF (i.e. ON, ICA, sellar/suprasellar structures)	AEA, PEA, ON, SOF
Bly [6]	2014	USA	5 heads + computer-aided modelling	TO	LRC	GSW removal between IOF and SOF	N/A	Lateral CS	IOF, SOF, ION
Bly [4]	2012	USA	4 heads +14 CT scans (computer-aided modelling)	2 TN, 8 TO	TN, LRC, TC, PC, SLC,	N/A	N/A	Pre - postchiasmatic cistern, CS, MC. SOF, Third ventricle, basal cistern, clivus	N/A
Ciporen [9]	2010	USA	5 heads	TO + TN + Supraorbital	PC (plus TN and supraorbital minicraniotomies)	N/A	N/A	PG, OC, cavernous ICA, clivus,	AEA, PEA, FES
Duz [24]	2009	Turkey	5 heads	TO + (TN + keyhole)	1) Inferolateral orbitotomy-EOA, 2) endoscopic endonasal medial orbital approach, and 3) transcranial keyhole endoscopic orbital approach	N/A	N/A	Orbit	TM, AEA, PEA

*ACF* anterior cranial fossa, *ACP* anterior clinoid process, *AEA* anterior ethmoidal artery, *ALC* anterior lacrimal crest*, CS* cavernous sinus*, EOA* endoscopic orbital approach, *FES* frontoethmoid suture, *FO* foramen ovale, *FR* foramen rotundum, *FS* foramen spinosum, *FZS* frontozygomatic suture, *GG* Gasserian ganglion, *GSW g*reater sphenoidal wing, *ICA* internal carotid artery, *ILTEA* inferolateral transorbital approach, *IOF* inferior orbital fissure, *ITF* infratemporal fossa, *KT* Kawase triangle, *LOW* lateral orbital wall, *LRC* lateral retrocanthal, *LSW* lesser sphenoidal wing, *LWCS* lateral wall of the cavernous sinus, *MC* Meckel`s cave, *MCF* middle cranial fossa, *MIS* middle incisural space, *MLA* meningolacrimal artery, *MMA* middle meningeal artery, *MOB* meningoorbital band, *MOW* medial orbital wall, *N/A* not applicable, *OA* orbital apex, *OC* optic canal, *ON* optic nerve, *PC* precaruncular, *PCF* posterior cranial fossa, *PEA* posterior ethmoidal artery, *PS* preseptal lower eyelid, *SF* supraorbital foramen, *SLC* superior eyelid crease, *SOF* superior orbital fissure, *ST* superior turbinate, *TL* temporal lobe, *TM* temporalis muscle, *TN* transnasal, *TO* transorbital, *VC* Vidian canal, *VN* Vidian nerve, *ZF* zygomaticotemporal foramen, *ZFB* zygomaticofacial bundle, *ZTB* zygomaticotemporal bund

### Indications

TEAs refer to a group of surgical corridors that reach or pass through the orbit without removing any part of the bony orbital rim or adjacent structures [3, 46, 47]. These approaches may be indicated for the treatment of pathologies located within or adjacent to the orbit [3, 31, 36, 43, 47]. They may also be used to target distant anatomical regions by using the orbital cavity as a corridor [1, 2, 4, 5, 8, 10, 14–16, 18, 19, 21, 25, 27, 29, 31, 33, 34, 37, 38, 40, 41, 47, 48, 56, 57]. They can be used either as a uniportal route [3, 5, 8, 14, 18, 27, 33, 36, 38, 47, 48, 50, 56] or may be combined with transnasal, transmaxillary, or supraorbital paths [1, 2, 4, 9–12, 16, 18, 20, 24, 28, 29, 31, 34, 41, 44, 46, 50–52, 58]. The decision making regarding the approach must be done considering the critical (neurovascular) structures involved by or adjacent to the pathology, the space needed for insertion of instruments, capability to reach the target from the approach angle, possibility to perform a reconstruction, corridor-related morbidity, and experience of the surgical team. The patients’ preference must also be taken into consideration [3, 8, 11, 14, 16, 18, 25–27, 29, 34, 46, 50, 52, 56, 57].

### Surgical techniques

**Fig. 3:**
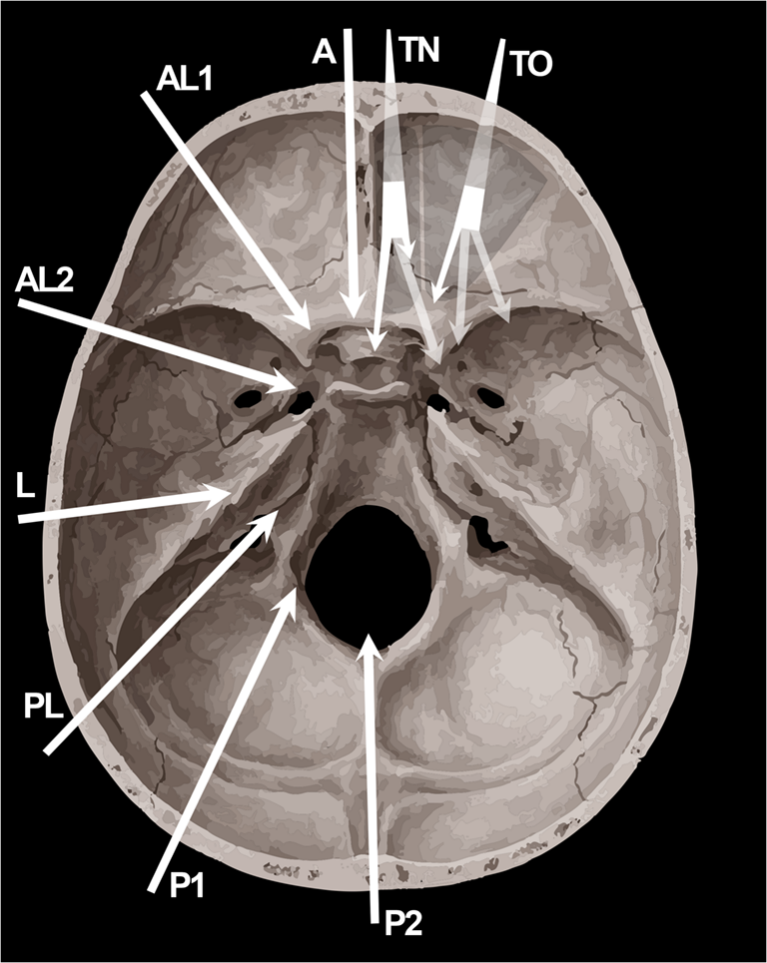
Scheme depicting the extension and reach of transorbital endoscopic approaches (TO) with respect to transnasal endoscopic (TN) and most relevant open skull base approaches. **A** Anterior approaches (e.g., subfrontal); AL1, paramedian anterolateral approaches (e.g., supraorbital); AL2, anterolateral approaches (e.g., pterional, frontotemporal, orbito-zygomatic, frontotemporal-orbitozygomatic); L, lateral approaches (e.g., transpetrous, subtemporal middle cranial fossa, infratemporal); PL, posterolateral (e.g., trans-sigmoid, retrosigmoid); P1, paramedian posterolateral approaches (e.g., far lateral); P2, posterior approaches (e.g., suboccipital) [17]

**Fig. 4:**
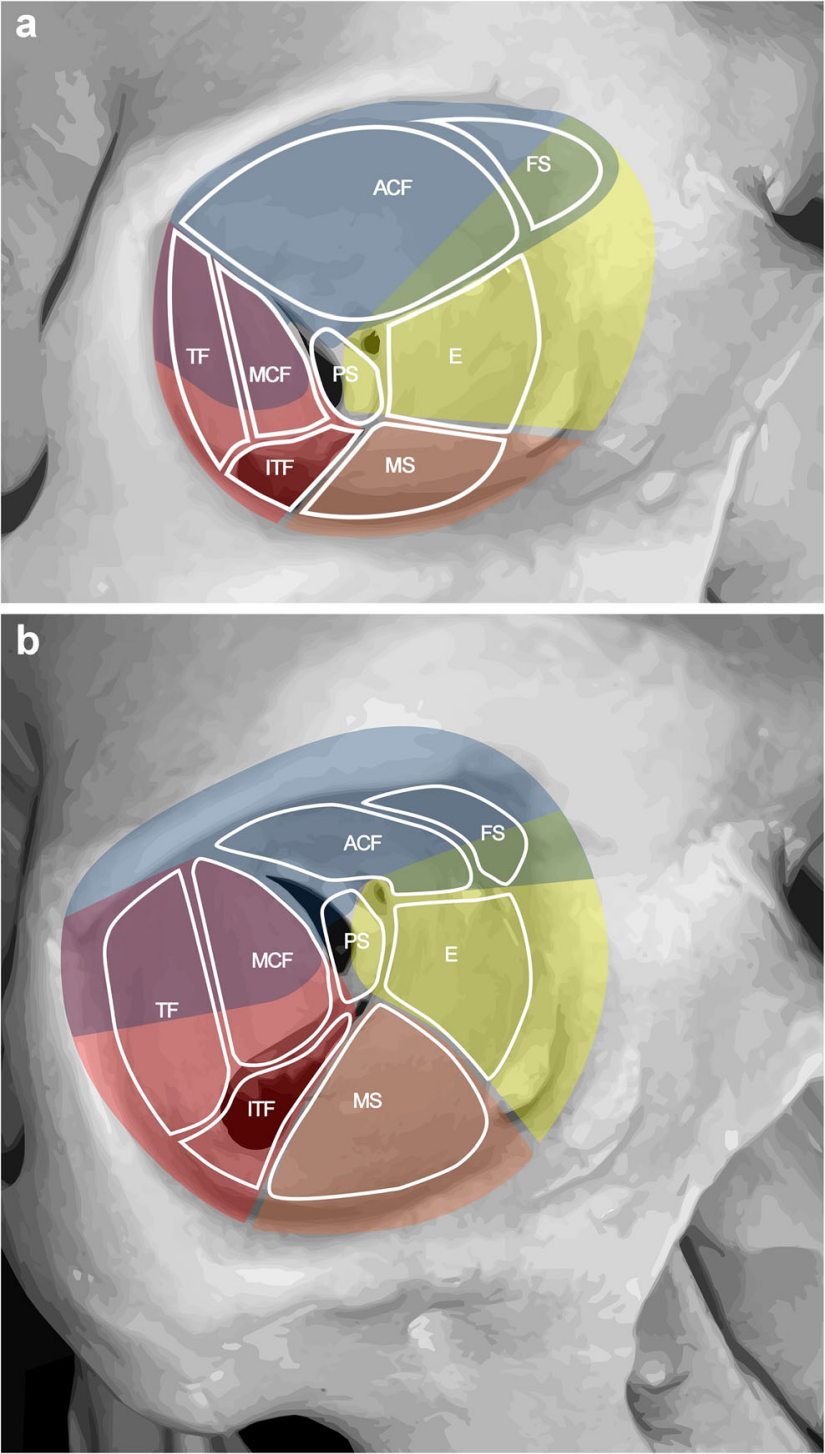
**A**, **B** Schemas presenting the relations of transorbital approaches with different anatomical sites. Right orbit of a dry skull. ACF, anterior cranial fossa, E, ethmoids; FS, frontal sinus; ITF, infratemporal fossa; MCF, middle cranial fossa; MS, maxillary sinus; PS, TF, temporal fossa. The colors indicate the transorbital surgical approaches as blue, superior eyelid; yellow, precaruncular; red, lateral retrocanthal; orange, inferior eyelid

**Fig. 5:**
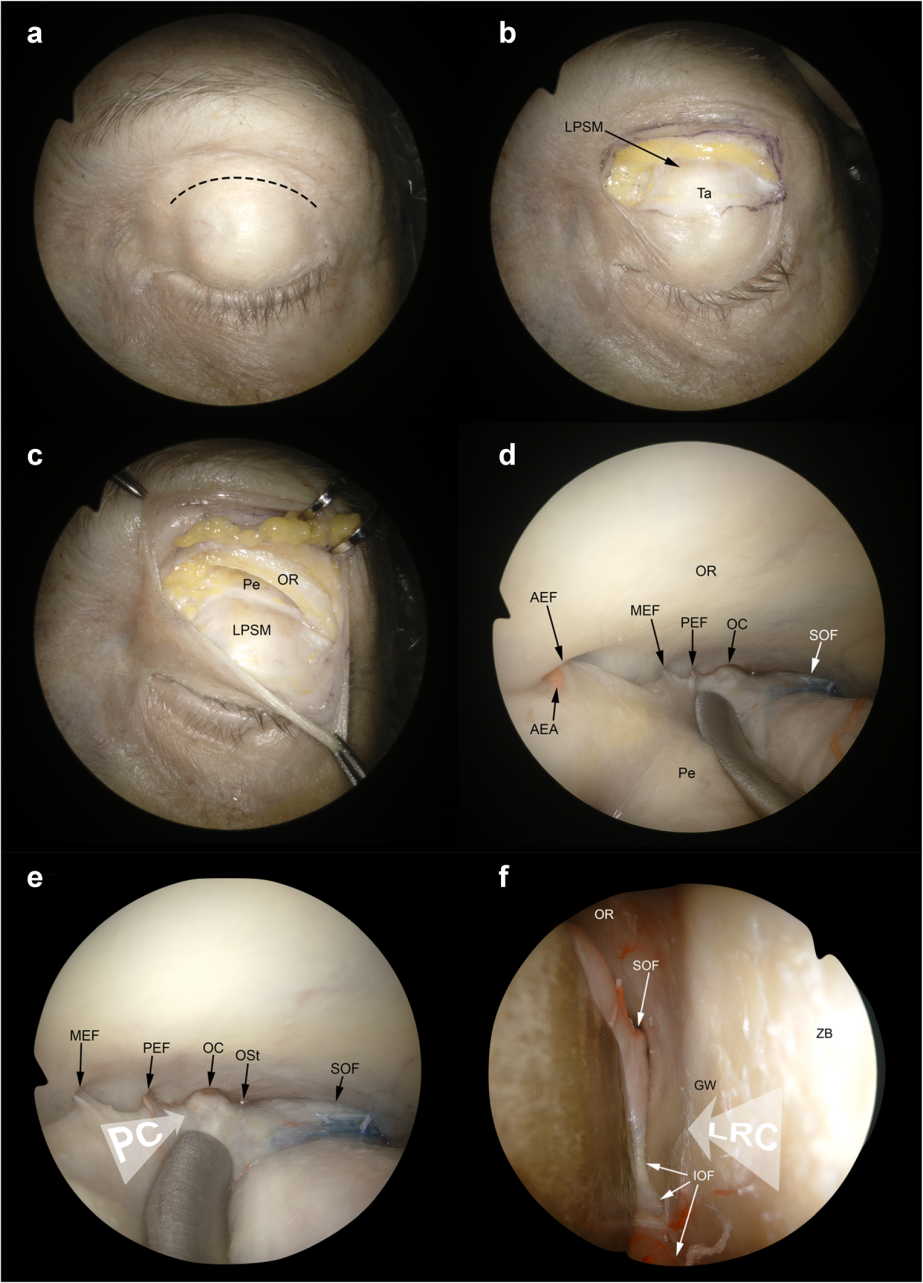
Superior eyelid and precaruncular approaches. **A** The superior eyelid crease approach starts with a skin incision performed at level of the supratarsal fold (*black dashed line*). **B** Superior tarsus (Ta) and levator palpebrae superioris muscle (LPSM) are identified. **C** The superior orbital rim is detached from the periorbit (Pe). **D** The subperiosteal dissection is continued along the orbital roof (OR). The anterior (AEF), medial—when present—(MEF), and posterior ethmoidal foramina (PEF) are identified in the medial aspect of the surgical corridor. The optic canal (OC) and the superior orbital fissure (SOF) are identified in the posterior portion of the orbit. **E** Both the precaruncular (PC) and lateral retrocanthal approach (LRC) display an overlap as regards the superior eyelid crease corridor. The trajectory of the precaruncular approach (*white arrow*, PC) lies at the medial aspect of the orbital cavity and requires sequential cut of the ethmoidal bundles. **F** The lateral retrocanthal approach (*white arrow*, LRC) is located in the lateral aspect of the orbital cavity and, similarly to the superior eyelid crease approach, offers direct exposure of the inferior orbital fissure (IOF), inferiorly, zygomatic bone (ZB) and greater sphenoidal wing (GW) laterally, and SOF superiorly. AEA, anterior ethmoidal artery; Ost, optic strut

**Fig. 6:**
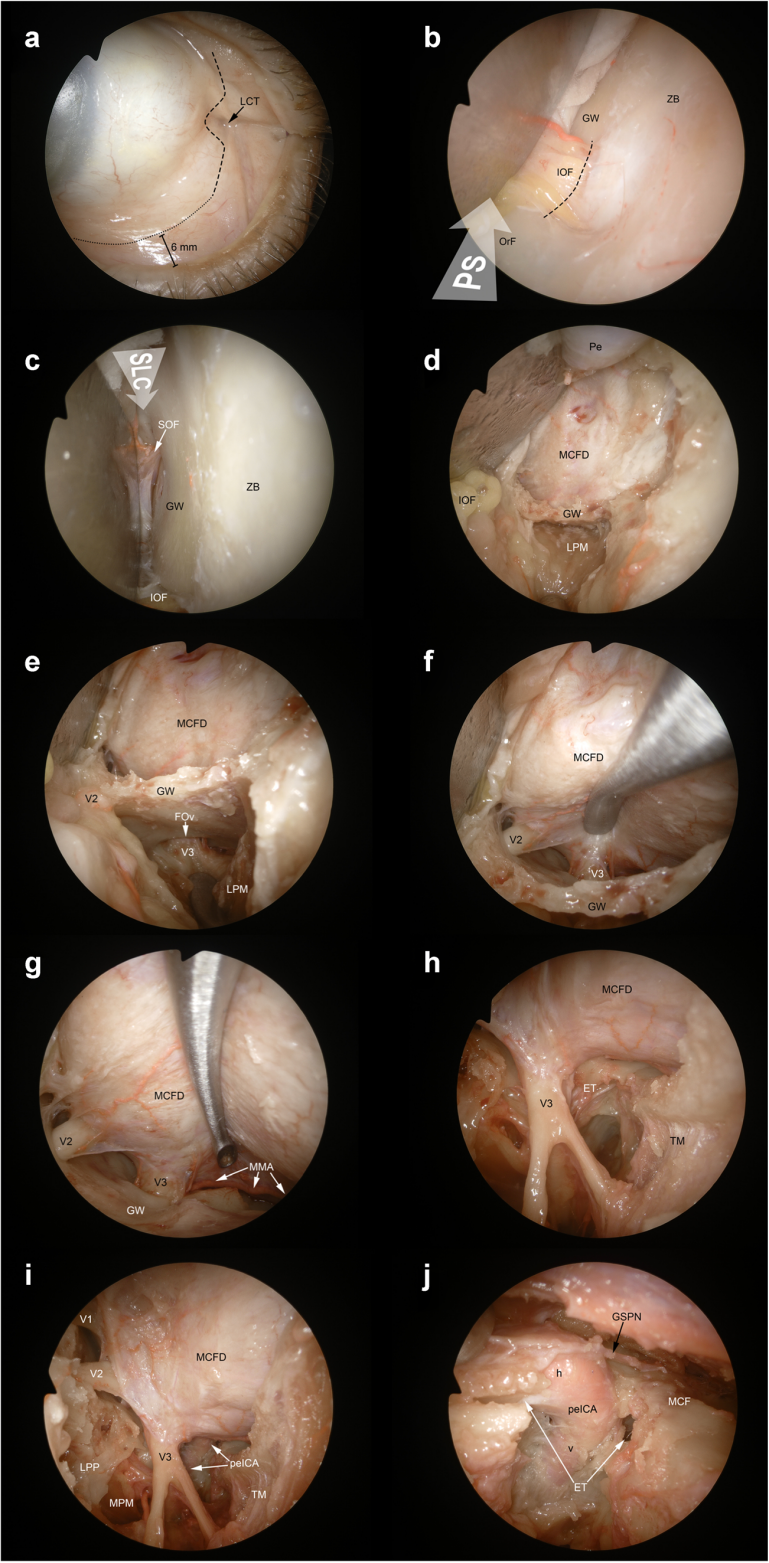
Lateral retrocanthal and preseptal lower eyelid approaches. **A** The lateral retrocanthal (LRC) approach starts with a conjunctival incision (*black dashed line*) of the palpebral conjunctiva located on the lateral aspect of the orbital rim and passing posterior to the lateral canthal tendon (LCT). With the aim of increasing maneuverability and exposure through the inferolateral orbital quadrant, the lateral retrocanthal approach can be combined with a preseptal lower eyelid approach (PS), which is also started with a conjunctival incision (*black dotted line*) on the inner surface of the lower eyelid. **B** The preseptal lower eyelid approach (*white arrow*, PS) exposes the orbital floor (OrF) and early stops at the inferior orbital fissure (IOF), which needs to be cut (*black dashed line*) to extend exposure to the greater sphenoidal wing (GW) while merging the inferior quadrant corridor with the lateral quadrant corridor (*i.e.* inferolateral transorbital endoscopic approach). **C** The lateral retrocanthal shares the potential to expose the greater sphenoidal wing and adjacent structures with the superior eyelid crease (SLC) approach (*white arrow*, SLC). **D** The removal of the coronal portion of the greater sphenoidal wing provides access to masticatory space, inferiorly, and middle cranial fossa dura (MCFD), superiorly. **E** The dissection can be continued along the extracranial aspect of the horizontal portion of greater sphenoidal wing by dissecting lateral pterygoid muscle (LPM) off the skull base. This maneuver provides exposure of the foramen ovale (FOv) and the extracranial tract of the mandibular nerve (V3) in the infratemporal fossa. **F** Epidural dissection along the anterior portion of the middle cranial fossa exposes the intracranial segments of maxillary (V2) and mandibular nerves. **G** Posterior and lateral to the mandibular nerve, the middle meningeal artery (MMA) runs from the foramen spinosum with a medial-to-lateral direction and provides vascular supply to the dura mater of this anatomical region. **H** After completing the removal of the bony contour of foramina ovale and spinosum and sectioning the middle meningeal artery, the bony-cartilaginous junction of the eustachian tube (ET) is identified. The eustachian tube crosses the mandibular nerve posteriorly and runs from superolateral to inferomedial. **I** The petrous segment of the internal carotid artery (peICA) is located posteriorly to the bony-cartilaginous junction of the eustachian tube. **J** After removing the eustachian tube and removing the anterior contour of the carotid canal, the vertical (v) and the horizontal (h) subtracts of the petrous portion of the internal carotid artery are visualized. GSPN, greater superficial petrosal nerve; LPM, lateral pterygoid muscle; LPP, lateral pterygoid plate; MPM, medial pterygoid muscle; MCF, middle cranial fossa; V1, ophthalmic nerve; Pe, periorbit; SOF, superior orbital fissure; TM, temporalis muscle; ZB, zygomatic bone

**Fig. 7:**
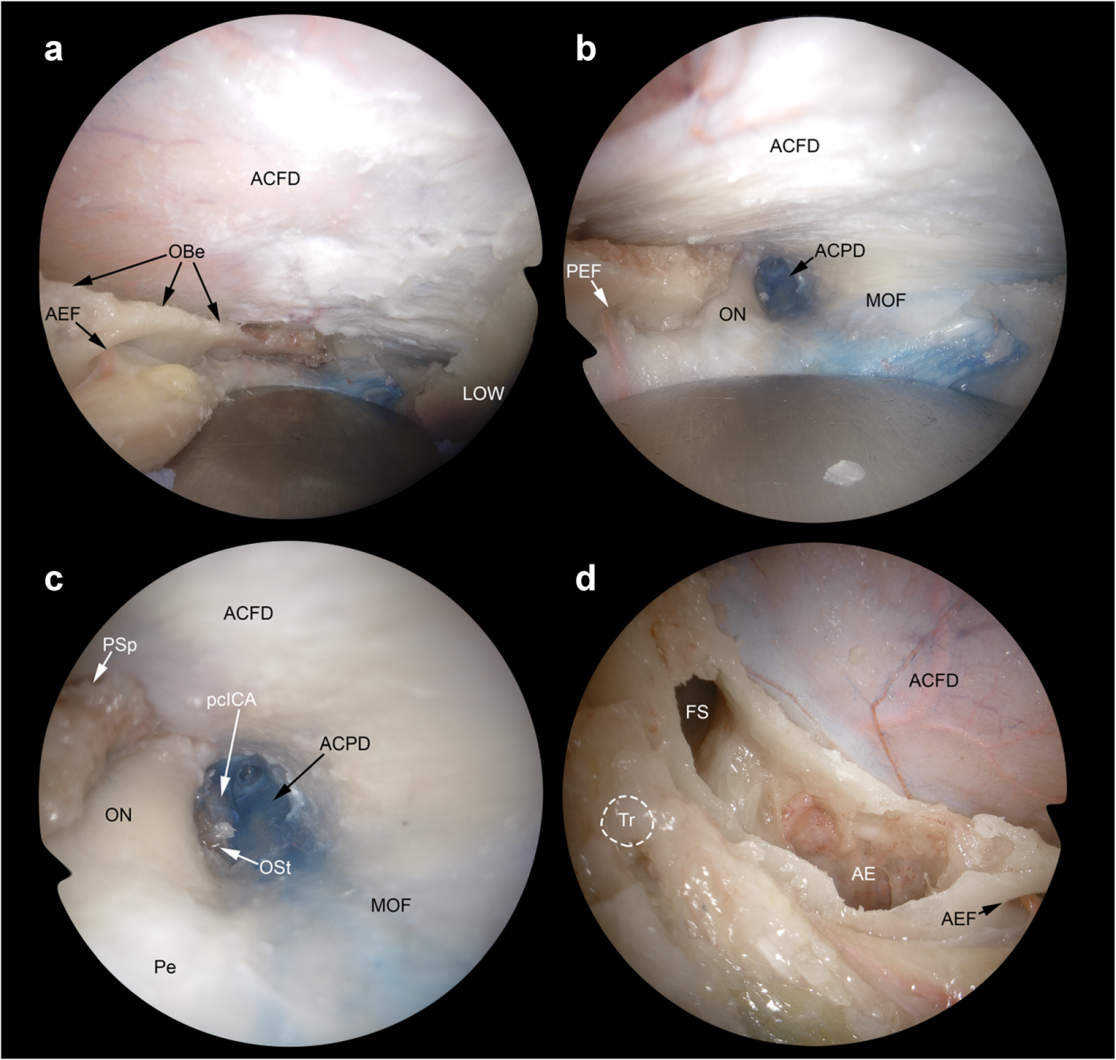
Transorbital exposure of the lateral anterior skull base. **A** The removal of the orbital roof provides exposure of the anterior cranial fossa dura (ACFD), which can be resected or incised to expose the inferior aspect of the frontal lobe and related neurovascular structures. Among transorbital endoscopic approaches, superior eyelid crease and precaruncular provide the best exposure of this portion of the cranial base and adjacent structures. The orbital beak (OBe) is the line located above ethmoidal foramina where the anterior cranial base turns from horizontal to cranially-convex (i.e*.*, from the ethmoidal roof to the orbital roof, respectively). The lateral orbital wall (LOW) can be used as landmark to define the lateral limit of the craniectomy. **B** Posterior craniectomy can include the anterior clinoid process, medially, and lesser sphenoidal wing, laterally. This provides exposure of the intracanalicular portion of the optic nerve (ON), anterior clinoid process dura (ACPD), and meningo-orbital fold (MOF), which is the area where the dura of anterior and middle cranial fossae turns into periorbit. **C** Focusing on the posteromedial portion of the surgical corridor, the optic strut (OSt) between the optic nerve and the paraclinoid tract of the internal carotid artery (pcICA). **D** In the most medial and anterior portion of the surgical corridor, bone removal of the superomedial orbital wall provide access to the frontal sinus (FS), frontoethmoidal region, and anterior ethmoid (AE). AEF, anterior ethmoidal foramen; Pe, periorbit; PSp, planum sphenoidale; PEF, posteror ethmoidal foramen; Tr with *white dashed line*, position of the trochlea

**Fig. 8:**
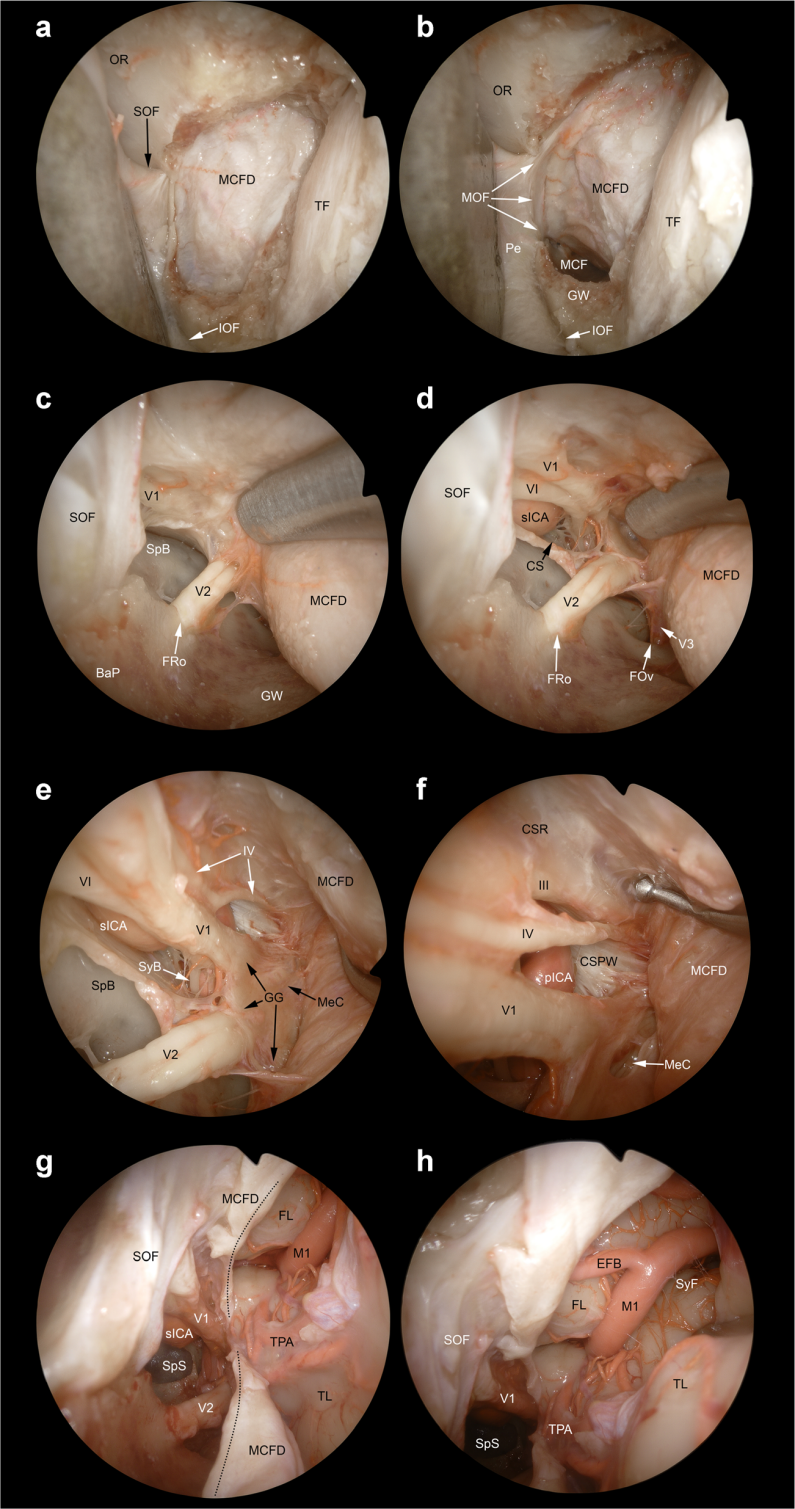
Transorbital exposure of the lateral middle skull base, parasellar area, and Sylvian fissure. **A** The middle cranial fossa dura (MCFD) can be exposed through a craniectomy in the area between the superior orbital fissure (SOF) and inferior orbital fissure (IOF). This portion of the skull base can be exposed through both the superior eyelid crease approach and lateral retrocanthal approach. The former provides a slightly descending trajectory towards the middle cranial fossa (MCF), whereas the latter route is parallel to the plane of the horizontal portion of the greater sphenoidal wing (GW). **B** The meningo-orbital fold (MOF) is identified as the line where the dura of the middle and anterior cranial fosse merge with the periorbit. **C** Epidural dissection along the middle cranial fossa allows exposure of the oftalmic (V1) and the maxillary (V2) branches of the trigeminal nerve, which run towards the superior orbital fissure and foramen rotundum (FRo), respectively. **D** Interdural dissection above the trigeminal branches provides access to the parasellar area and allows identification of the mandibular branch of the trigeminal nerve (V3) and foramen ovale (FOv). The cavernous sinus (CS) is identified above the ophthalmic nerve (i.e*.*, infratrochlear or Parkinson’s triangle) and in the space between the ophthalmic and maxillary nerves (i.e*.*, anteromedial or Mullan’s triangle) and the parasellar tract of the internal carotid artery (sICA), abducens nerve (VI), and trochlear nerve (IV) are exposed. **E** Further posterior interdural dissection exposes the Gasserian ganglion (GG) and Meckel’s cave (MeC). **F** The oculomotor nerve (III), cavernous sinus roof (CSR), posterior wall of the cavernous sinus (PWCS), and paraclival portion of the internal carotid artery (pICA) can be identified by further elevating the dura propria of the parasellar area. **G** The dura propria of the parasellar area and lateral middle cranial base is incised (*black dotted line*) to access the intradural compartment Sylvian fissure (SyF). **H** The first tract of the middle cerebral artery (M1), early frontal branch (EFB), and temporal polar arteries (TPA) are identified between the frontal (FL) and temporal lobe (TL). BaP, base of the pterygoid process; OR, orbital roof; Pe, periorbit; SyB, sympathetic branch of the abducens nerve; SpB, sphenoid body; TF, Temporal fossa

**Fig. 9:**
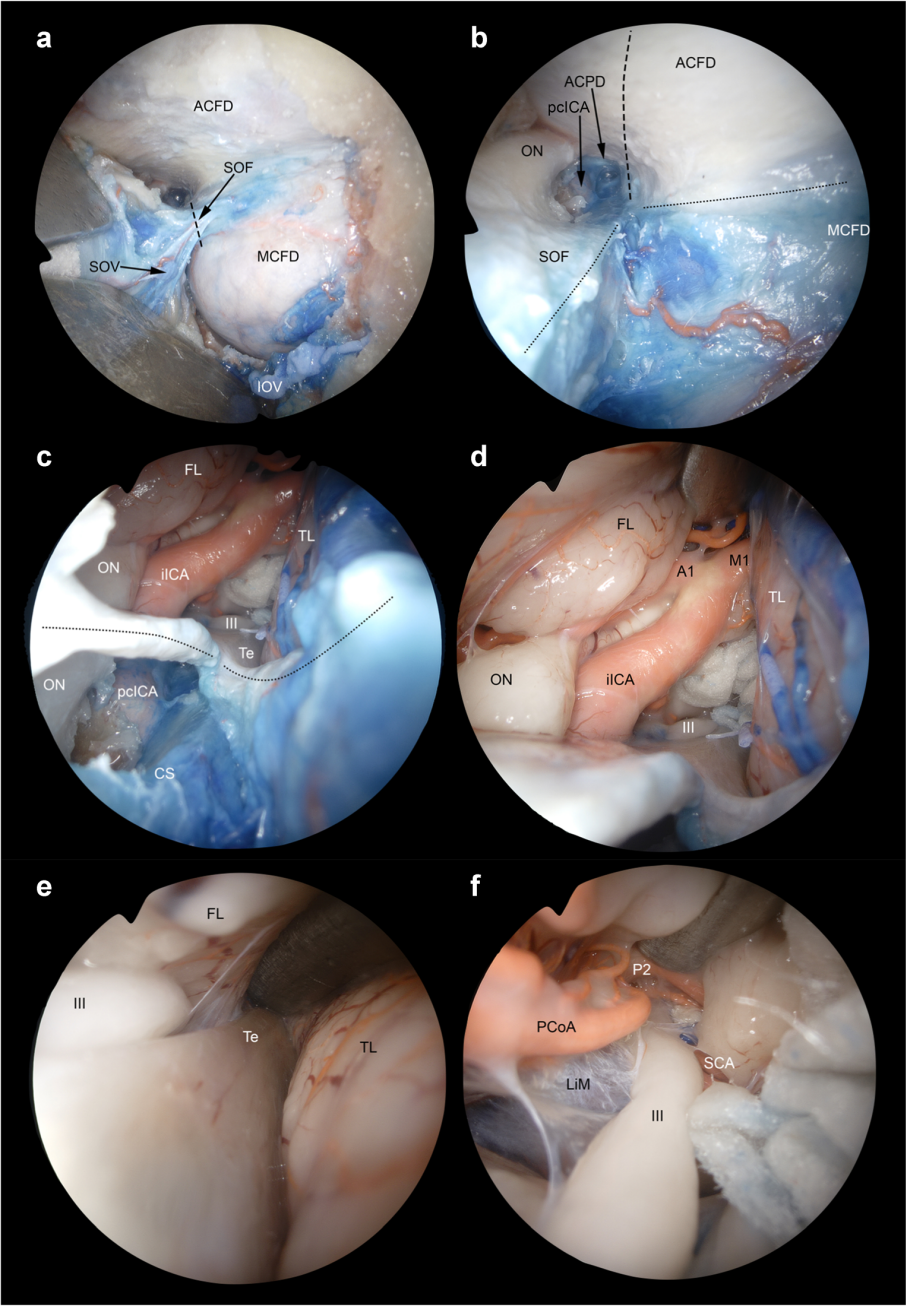
Extended transorbital transdural approach to the internal carotid artery bifurcation and adjacent structures. **A** The corridors through the dura of the anterior (ACFD) and middle cranial fossa (MCFD) can be merged by sectioning the meningo-orbital fold (*black dashed line*) in the lateral portion of the superior orbital fissure (SOF), which, as opposed to the medial portion, does not contain relevant neurovascular structures other than the superior ophthalmic vein (SOV) and meningo-orbital/-lacrimal or recurrent meningeal branch of the middle meningeal artery. **B** After sectioning the meningo-orbital fold (*black dotted line*), the full exposure of the dura of the posterolateral anterior cranial fossa, parasellar area, and lateral middle cranial fossa is achieved. Anterior clinoid process dura (ACPD), optic nerve (ON) and the paraclinoid tract of the internal carotid artery (pcICA) are identified. Among other potential transdural targets, the intracranial tract of the internal carotid artery (iICA) and its bifurcation can be exposed by incising the anterior cranial fossa and anterior clinoid process dura (*black dashed line*). This maneuver would be more difficult if the meningo-orbital fold is not sectioned. **C** Once the dura of the anterior clinoid process is incised, the following structures are encountered: oculomotor nerve (III), frontal lobe (FL), intracranial tract of the internal carotid artery, optic nerve, tentorium cerebri (Te), and temporal lobe (TL). Thanks to its tangential trajectory as respect to the anterior cranial fossa dural plane, the transorbital perspective provides the exposure of both the intradural and extradural portion of several neurovascular structures. Particularly, the figure shows both the intracranial and paraclinoid tract of the internal carotid artery as well as intradural and intracanalicular portions of the optic nerve. **D** The bifurcation of the internal carotid artery into the precommunicating tract of the anterior cerebral artery (A1) and proximal tract of the middle cerebral artery (M1) can be identified by moving the scope forward through the transdural window. **E**, **F** The free edge of the tentorium cerebri can be used as landmark to follow the oculomotor nerve (III) towards the ambiens cistern and interpeduncular fossa. The Liliequist’s membrane (LiM), postcommunicating tract of the posterior cerebral artery (P2), posterior communicating artery (PCoA), and superior cerebellar artery (SCA) can be also identified. CS, cavernous sinus; IOF, inferior ophthalmic vein

Transorbital endoscopic surgery is based on 4 pillar-approaches through orbital quadrants: the superior eyelid crease (SLC), precaruncular (PC), the lateral retrocanthal (LRC), and the preseptal lower eyelid (PS), which cross the superior, medial, lateral, and inferior orbital quadrants, respectively (Figs. 3, 4, 5, 6, 7, 8, and 9) [47]. Several variants of these pillar approaches have been described over the last decade in clinical and anatomical studies, each aiming to facilitate surgical goals [8, 9, 20, 24, 25, 29, 33, 44, 50–52, 59]. A thorough understanding of the anatomy of the eyelid is essential in each approach, and possible need for a reconstruction and additional corridors (i.e., multiportal approach) must also be precisely planned before surgery.

#### Superior eyelid crease approach

With the superior eyelid crease approach (Fig. 5), also named as upper eyelid approach, the superior orbit, frontal sinus, supraorbital and posterior-central portions of the anterior cranial fossa (ACF) and anterior skull base (ASB), and lateral portion of the middle cranial fossa (MCF) can be reached. This is the commonest approach used in transorbital endoscopic surgery [2–4, 8, 12, 15, 16, 18–21, 28, 29, 31, 32, 34, 36, 38, 47, 48, 51, 53, 56, 59]. The skin incision is made in the supratarsal fold as done in an upper blepharoplasty and can be tailored according to the path-to-target analysis [26, 47]. Deep to the preseptal orbicularis oculi muscle, the orbital septum is identified, through which the prelevator fat can be seen. Dissection is continued raising the deep surface of the orbicularis muscle towards the superior orbital rim. This is crucial to avoid the aperture of the orbital septum and periorbit, which causes fat to prolapse into the surgical corridor. After the identification of the orbital rim, the periosteum is incised, and dissection is further progressed in a subperiosteal plane through the orbital roof. In the posterior portion of the orbit, the orbital end of the optic canal is identified, and medially the ethmoidal foramina can be visualized. According to the target, the dissection can be extended as far laterally as necessary. Identification of the superior orbital fissure (SOF) is paramount to achieve adequate orientation in the most posterior and lateral aspect of the corridor. If a more lateral corridor is needed, detachment of lateral canthal ligament can be done [26]. Diamond burr or a chisel is used to perform the craniectomy, which is sized and located depending on the targeted area [16, 37, 56]. The dura of the ACF and MCF can be elevated and/or incised based on the situation of the disease.

#### Precaruncular approach

This approach via the medial quadrant provides a direct and avascular access to medial orbital roof, lamina papyracea, ethmoidal arteries, cavernous sinus, parasellar and paraclinoid tracts of the internal carotid artery, optic nerve, and the central corridor towards the anterior skull base (ASB) [3, 4, 9, 10, 26, 36, 46–48, 58].

An incision between the caruncle and skin is made through the conjunctiva at the apex of the medial canthus. The avascular plane is entered deep to the Horner’s muscle and to the posterior limb of the medial canthal tendon, and the periorbit is incised at crista lacrimalis (i.e., the posterior border of the lacrimal fossa). Dissection is performed from anterior to posterior between the periorbit and the medial orbital wall. The level of the ASB can be estimated by the ethmoidal bundles which are found along the fronto-ethmoidal suture and can be cauterized and cut. Reaching the posterior ethmoidal artery warns the surgeon that the optic nerve is close (i.e., around 7 mm posterior to the posterior ethmoidal foramen) and attention must be paid not to damage it (Fig. 5). After that level, dissection through the medial orbital wall makes the surgeon reach the orbital apex and then the bony removal is performed depending on the anatomical site of the target [26, 47].

#### Lateral retrocanthal approach

Via LRC approach, access to the deep lateral orbit, lateral aspect of the ACF, MCF, and infratemporal and temporal fossa is possible [3, 4, 28, 40, 47]. LRC overcomes morbidities like scarring and disruption of eyelid support caused by cutaneous or canthotomy incisions. A conjunctival incision is performed immediately posterior to the insertion of the lateral canthus (Fig. 6). The subperiosteal dissection is performed along the lateral orbital wall, from the inferior orbital fissure (IOF) to the orbital roof. This maneuver exposes the greater sphenoidal wing (GSW) (located below the SOF, above and lateral to the IOF, and posterior to the zygomatic bone), whose removal provides access to the temporal fossa, infratemporal fossa, and MCF [47]. The optic nerve is not at risk through this corridor as it stands medially in the orbit and is separated from the lateral wall by the contents of the SOF. At the superior aspect of the lateral orbital wall lies the sphenofrontal suture by which a superior craniectomy yields to lateral frontal fossa, while an inferior craniectomy guides to the MCF [47].

#### Preseptal approach

The PS approach is useful to access to the inferior orbit [38, 47]. It can be combined with LRC or PC to increase maneuverability and exposure of the lateral and medial orbital quadrants, respectively (Fig. 6). It gives a path through orbital floor, maxillary sinus, IOF, and foramen rotundum. The conjunctival incision for a preseptal approach is made 2 mm inferior to the tarsus on the conjunctival surface of the lower eyelid (6 mm inferior to the eyelid margin). The orbicularis oculi muscle is identified, and the dissection is carried out on its posterior face, which is anterior to the inferior orbital septum. The orbicularis muscle is followed through the inferior orbital rim, where the periosteum is incised and lifted off the orbital floor. Dissection may proceed further by sectioning the infraorbital bundle and IOF [47].

#### Extended and combined transorbital endoscopic approaches

Along with the 4 pillar TEAs, extended or combined approaches can be applied in selected cases [2, 4, 5, 8–12, 16, 18, 20, 24, 25, 28, 29, 31, 34, 40, 41, 44, 50–52]. In the literature, TEAs are mostly used in combination with transnasal endoscopic procedures [1, 2, 4, 9–12, 22, 24, 28, 29, 34, 40, 41]. Other than transnasal corridors, different additional extraorbital corridors, such as supraorbital, lateral orbital, and pterional, have also been investigated and described in combination with transorbital routes (Figs. 7, 8, and 9) [9, 16, 20, 44, 51, 52]. Transorbital extended approaches, in which more than one orbital quadrant was used, have also been presented [25, 59].

#### Endoscopic transorbital craniectomy and bony landmarks

The term craniectomy describes the removal of the bone cover on the dura with no bone repositiong at the end of the procedure [26]. The type of craniectomy is determined by the anatomo-surgical target for the underlying pathology. A drill, ultrasonic dissector, or chisel can be used to remove the bone at the desired site [47]. Surgeon should be familiar with the bony landmarks when performing a craniectomy. The thickness of bone varies throughout the orbital walls and different landmarks need to be exposed to complete the bone removal safely and accurately. In the 33 studies in which the craniectomy was mentioned in the text, SOF was the most common landmark (75.8%), followed by the IOF (54.5%) and GSW (39.3%). The other anatomical structures that were pointed as landmarks were temporalis muscle (TM), lateral orbital wall (LOW), medial orbital wall (MOW), anterior ethmoidal artery (AEA), posterior ethmoidal artery (PEA), lesser sphenoidal wing (LSW), meningo-orbital band (MOB), frontozygomatic suture (FZS), anterior clinoid process (ACP), optic nerve (ON), optic canal (OC), internal carotid artery (ICA), foramen ovale (FO), foramen rotundum (FR), foramen spinosum (FS), vidian canal (VC), vidian nerve (VN), zygomaticotemporal foramen (ZF), supraorbital foramen (SF), frontoethmoidal suture (FES), zygomaticofacial bundle (ZFB), zygomaticotemporal bundle (ZTB), orbital apex (OA), anterior lacrimal crest (ALC), and meningolacrimal artery (MLA) [1, 2, 4, 5, 8–16, 18–20, 24, 25, 27–29, 32–34, 37, 38, 40, 41, 44, 47, 51–53, 56, 58].

### Reconstruction

As in general in SB surgery, reconstruction may be necessary to achieve a safe separation between different compartments (i.e., sinonasal tract, orbit, intracranial space). Postoperative cerebrospinal fluid (CSF) leak is a common concern related to TEAs and the surgeon must be familiar with different reconstructive techniques. In case of small defects with limited CSF leak, reconstruction is deemed unnecessary thanks to the support of the orbital structures [1, 14, 56]. For larger defects, reconstruction is indeed required, and the technique of reconstruction has to be tailored according to pathology, type of approach, and craniectomy size and site. When the defect is extended beyond the limits of the orbit (e.g., frontal sinus, cribriform plate, planum sphenoidale, lateral recess of the sphenoid), watertight closure should be achieved following the principles of transnasal endoscopic SB reconstruction [3, 41, 47, 48, 58]. Various materials such as autologous grafts (e.g., fascia lata, temporalis fascia, iliotibial tract, abdominal fat, septal mucoperichondrium) or synthetic materials (e.g., TachoSil®, AlloDerm®, DuraGen®) have been used either alone or in combination [8, 12, 14, 16, 28, 29, 31, 32, 34, 38, 40, 41, 43, 48, 53, 58]. In the majority of the studies, a multilayer reconstruction was conducted. A total of 74 patients required dural reconstruction, and postoperative CSF leak was reported in 3 (4.1%) [8, 12, 15, 16, 28, 29, 31, 32, 34, 40, 41, 48, 53, 58]. Even after the reconstruction of large defects, the risk of CSF leak appears to be low as the orbital contents keep the reconstruction in place and tight [1, 12, 14, 46, 48, 56]. Pulsation of the globe may be noted for 1 to 2 weeks postoperatively, which generally resolves spontaneously [8, 56, 60]. In addition, in order to prevent postoperative enophthalmos, Medpor can be placed over the dural defect as a buttress [29]. When the lateral orbital rim was removed in extended TOA, the orbital rim can be reconstructed by a mini plate or a titanium mesh [29, 38].

### Complications

TEAs provide the chance to avoid some complications related to open craniotomies. However, by nature, they also have risks of complications. A total of 60 complications were reported in 193 cases presented (Tables 3 and 4) [3, 8, 12, 15, 16, 28, 29, 31, 34, 53, 58]. The majority of the complications reported were transient. Balakrishnan et al. [3] reported 3 persistent vision loss after surgery (3% of cases), Golbin et al. [28] reported one persistent abducens nerve paresis, and Lee et al. [34] reported postoperative facial numbness in 38.1% of their patients which did not resolve. When the complications were evaluated in terms of Clavien-Dindo classification, all except one were Grade 1 and 2 [23]. The only complication requiring surgical intervention (Grade 3b) was an orbital pseudomeningocele which resolved after shunt insertion [8]. Table 5 shows the Clavien-Dindo classification for grading complications [23].

**Table 3:** Complications of transorbital approaches in which surgical procedures were presented (numbers of complications) (superscripts in the first row indicate the reference numbers)

Author	Number of TO cases	Complications
Jeon [29]	9	Complete ptosis improved in 6 months (1), mild ptosis (3)
De Rosa [16]	1	Proptosis which resolved in 6 months (1)
Lee [34]	9	Decrease in visual acuity (1), CN V neuropathy (2), CN VI neuropathy (2), ptosis (3) keratitis (2)
Golbin [28]	12	Transient CNV_1_ hypoesthesia (2), transient ptosis (1)
Kong [31]	18	CSF leaks (2), transient lateral rectus muscle paresis (2), transient ptosis (3)
Dallan [15]	14	Upper eyelid necrosis (1), diplopia (3), CNV_2_ hypoesthesia (3), CNV_1_ hypoesthesia (1), palpebral edema (3) (of which 1 persistent)
Chen [8]	2	Orbital pseudomeningocele (1)
Dallan [12]	4	Superior eyelid edema (2)
Balakrishnan [3]	107	Diplopia (14), persistent vision change (3)
Park [53]	11	CSF leak (1), diplopia (1), ptosis (1)
Raza [58]	6	Diplopia (1)
Total Number	193	60 (31.1%)

**Table 4:** Rates of complications. * The rate of CSF leak in patients undergoing dural defect reconstruction is 4.1% (superscripts in the second row indicate the reference numbers)

Complication	Number of cases	Single-series rate	Overall rate in complications	Overall rate in total cases
Diplopia	23[3, 15, 31, 53, 58]	9.1-22.2%	38.3%	11.9%
Ptosis	12 [28, 29, 31, 34, 53]	8.3-33.3%	20%	6.2 %
Proptosis	1 [16]	100%	1.6%	0.5%
Palpebral edema	5 [12, 15]	21.4-50%	8.3%	2.5%
CSF Leak	3 [31, 53]	9-11.1%	5%	1.5%*
Vision change	4 [3, 34]	2.8-11.1%	6.6%	2%
Orbital pseudomeningocele	1 [8]	50%	1.6%	0.5%
Keratitis	2 [34]	22.2%	3.3%	1%
Trigeminal nerve neuropathy	8 [15, 28, 34]	16.6-28.5%	13.3%	4.1%
Upper eyelid necrosis	1 [15]	7.1%	1.6%	0.5%

**Table 5:** The Clavien-Dindo Classification of Surgical Complications [56]

Grade	Description
Grade 1	Any deviation from the normal postoperative course not requiring surgical, endoscopic or radiological intervention. (Allowed therapeutic regimens are: drugs as antiemetics, antipyretics, analgesics, diuretics, electrolytes, and physiotherapy. This grade also includes wound infections opened at the bedside)
Grade 2	Requiring pharmacological treatment with drugs other than such allowed for grade I complications Blood transfusions and total parenteral nutrition are also included
Grade 3	Complications requiring surgical, endoscopic or radiological intervention Grade 3a—intervention not under general anesthetic Grade 3b—intervention under general anesthetic
Grade 4	Life-threatening complications; this includes central nervous system complications which require intensive care Grade 4a—single-organ dysfunction (including dialysis) Grade 4b—multi-organ dysfunction
Grade 5	Death of the patient

## Discussion

TEAs, initially described as ancillary alternatives to traditional transcranial/transnasal routes, have been evolving to the state of well-established surgical methods, which are intended to overcome the limits of conventional procedures for selected pathologies of the SB, either alone or in combination with other techniques [12, 39, 47]. For this reason, they raised a progressively increasing interest, as evident from the number of publications in a relatively short timeframe (Fig. 2). They have become relevant tools in SB surgery, facilitating access to a number of sites deemed challenging to reach, yet with relatively low morbidity [39, 41, 47].

In order to achieve the surgical goal, the surgical approach must be precisely selected, which majorly depends on the quadrant of the orbit that is involved by or is the most forthright route to the target pathology [46, 47]. The basic requirement to safely harvest a transorbital surgical corridor and properly manipulate the lesion is sound knowledge of surgical anatomy. As learnt from transnasal endoscopic surgery, this should ground on the understanding of anatomical landmarks, which are basic anatomical relationships through which the surgeon can maintain orientation even in an intricate surgical corridor. The present paper systematically summarizes the main landmarks that were emphasized throughout available publications (Tables 1 and 2; Figs. 3, 4, 5, 6, 7, and 8). The created pathway through the target needs to give the surgeon optimal visualization and provide space for the use of endoscopic instrumentation. This is crucial both for the manipulation of the target and SB reconstruction. By TEAs, this short and direct path yielding to the needed comfortable working space can be frequently achieved, but one should keep in mind the spectrum of potential surgical alternatives to offer the best treatment to patients. The flexibility that the SB surgeon/team has in hand, which is the ability to extend or combine various approaches, provides a significant comfort and success in “complex” procedures [26, 39, 46, 47]. The surgeon can benefit from the possible better exposure provided by a combination of approaches. In a recent study, it was indicated that the endoscopic transnasal approach better exposes the inferomedial 1/3 of the SOF and the cavernous sinus while the TEA yields a better exposure of the superolateral 2/3s of the SOF. A combination of the two approaches can make it feasible to access the entire SOF endoscopically [35]. In another study, the combined transnasal-transorbital approach to the petrous apex was investigated in cadavers in order to quantify the amount of bone removal that can be obtained via each pathway, and the authors concluded that with a combined approach, 97% of the bone removal can be performed [61].

The application of these procedures is best set by close collaboration between surgeons from different specialties such as otorhinolaryngology, neurosurgery, and ophthalmology. The SB surgeon can be unfamiliar with transorbital procedures. There doubtlessly exists a learning curve which can best be surpassed by proper anatomical training and teamwork [2, 39]. The proper instrument setting is also important for performing this type of surgery which has to include an oculoplastic set with retractors, corneal protectors, lacrimal probes, a SB endoscopic set with high-quality endoscopes (0, 30, 45 and 70 degrees), drill, endoscopic Doppler probe. and surgical navigation system [46]. Dedicated instruments specifically designed for this type of surgery are worth designing and testing in order to overcome pitfalls like orbital fat blocking the view. The surgical team always needs to keep in mind that there may be need to intraoperatively shift the approach from endoscopic to an open one, and the preoperative planning must include every possible scenario on which the patient should be clearly informed and consented [26, 39].

The TEAs have been applied to various anatomical sites of the SB in several clinical studies. Orbital cavity and adjacent portions of the ASB and MSB are the most frequently targeted areas. Other potential targets have been recently analyzed and discussed. Gerges et al.[27] investigated the application of TEA for the ITF and PPS in anatomical specimens. They also presented a case with a recurrent glioblastoma of the ITF, which was approached through TEA for a biopsy. The authors conclude that TEA can be a safe alternative with less morbidity in this area, adding that pathologies extending inferiorly to the masticator space and neck may be a contraindication but those that extend to the MCF and spread anteriorly with orbital extension are more susceptible to this approach. Lee et al. [34] indicated that access to the CS via TEA may be considered more practical than the endoscopic transnasal approach. The reason of this provision is explained by the shorter access route, ability to perform interdural dissection through the CS, ability of facilitating exploration through the anteromedial (between V1 and V2) and anterolateral (between V2 and V3) corridors, and avoidance of morbidity caused by transnasal and/or open approaches. The authors however indicate that the TEA is a challenging route for the posterolateral triangle (corridor between the V3 and the petrous apex). They presented a case in which TEA was insufficient for approaching a dumbbell-shaped schwannoma involving the MCF and PCF who later required additional retrosigmoid surgery. Chen et al. [8] performed surgeries via TEA in two patients for lesions (gliosis) in the temporal lobe. They expressed that the TEA minimized risks related to conventional approaches to the temporal lobe like cognitive deficits, hemiparesis, cranial nerve deficits, and visual field loss by providing a direct path towards the temporal lobe and allowing earlier visualization of the target. It is also mentioned that by the TEA, the surgeon achieves an early visualization of the cranial nerves which may facilitate the avoidance of an injury. Studies indicate that the TEAs provided optimal exposure and surgical freedom for adequate handling of lesions of the ACF and MCF [3, 16, 47]. If needed, removal of the superior orbital wall is possible, exposing the entire ACF from the midline to its most lateral point. Suchlike, the removal of the lateral orbital wall may help for an adequate view of MCF, from the lateral wall of the cavernous sinus to the lateral aspect of the temporal lobe [51].

TEAs have been successfully applied in various pathologies ranging from CSF leak to SB tumors (Table 6) [3, 8, 12, 16, 27, 28, 32, 36, 40, 43, 47, 53, 58]. Among tumors, with 67 (65.6%) presented cases, meningioma was the most common pathology followed by trigeminal schwannomas (9.8%). In the 102 tumor cases presented, 50 (49.0%) gross-total resections, 9 (8.8%) near-total resections, 30 (29.4%) sub-total resections, and 6 (5.9%) partial-resections were reported [12, 15, 16, 27, 29, 31, 32, 34, 53, 58]. In 7 (6.9%) patients, biopsies were obtained through TEAs [27, 28]. Radiation therapy was performed with different techniques (stereotactic radiotherapy, Gamma Knife radiosurgery, proton beam radiotherapy) in 19 cases in whom noncomplete resection was achieved [28, 31, 34]. Inflammatory pathologies like epidural abscesses, frontoorbital mucoceles, and cavernous sinus thrombosis were also managed by TEAs either alone or in combination with transnasal approach [3, 28, 36, 47].

**Table 6:** Pathologies and clinical conditions for which transorbital endoscopic surgeries were applied in the literature

Pathologies and clinical conditions	Number of cases	References
Meningioma	67 (45.0%)	[12, 15, 16, 28, 29, 31, 32, 34, 40, 53]
Schwannoma	10 (6.7%)	[12, 29, 31, 34]
Dermoid cyst	2 (1.3%)	[29, 34]
Chondrosarcoma	2 (1.3%)	[29, 34]
Osteoblastoma	1 (0.7%)	[28]
Osteosarcoma	1 (0.7%)	[31]
Gliosis	2 (1.3%)	[8]
Inflammation/infection/abscess	17 (11.4%)	[3, 36, 47]
CSF Leak	23 (15.4%)	[3, 41, 47, 48, 58]
Plasmocytoma	1 (0.7%)	[31]
Teratoma	1 (0.7%)	[31]
Glioblastoma	1 (0.7%)	[27]
Metastatic tumor	2 (1.3%)	[28, 29]
Mucocele	7 (4.7%)	[26, 36, 47]
Hemangioma	1 (0.7%)	[43]
Cavernous sinus thrombosis	1 (0.7%)	[36]
Sebaceus gland carcinoma	1 (0.7%)	[31]
Malignant peripheral nerve sheat tumor	1 (0.7%)	[28]
Pituitary adenoma	1 (0.7%)	[32]
Adenoid cystic carcinoma	1 (0.7%)	[47]
Juvenile nasopharyngeal angiofibroma	1 (0.7%)	[58]
Olfactory neuroblastoma	1 (0.7%)	[58]
Paget disease	1 (0.7%)	[58]
Pseudotumor	3 (0.7%)	[28]
TOTAL	149	

With either small or invisible incisions, TEAs provide a pleasant cosmetic outcome. However, the absence of a surgical scar may give a misperception that the procedure is a minor surgery. The complications related to the globe, as well as neurovascular structures and eyelid apparatus, should always be kept in mind and the risks should be counseled with the patient (Tables 3 and 4). Care must be taken to protect the cornea and the globe intraoperatively as continuous orbital retraction may cause an increase of orbital pressure. This may also result in cardiac arrhythmias, thus, frequent monitoring of pupil size and blood pressure, along with electrocardiography, is required [29]. Intermittent relaxation of the eyeball every 20–30 min during the procedure and keeping tissues dislocation less than 10 mm is recommended [5, 48, 51].

Overall, TEAs have relatively less morbidity than traditional SB procedures. The reports indicate mostly minor morbidities, rapid postoperative healing, minimal pain, and short time of hospitalization [26, 39, 46, 47]. Despite these relatively low morbidity rates, it is the authors’ opinion that it is still too emphatic to name them as minimal invasive approaches, as complications do occur in a non-negligible rate of patients (31.1%) [3, 14, 34]. Indeed, the classification employed in this systematic review rates complications such as diplopia and upper eyelid necrosis as minor events, whereas one should take into consideration the dismal impact of these occurrences on patient’s quality of life. In fact, morbidity should not be considered as a secondary issue, as most patients receiving a TEA are affected by benign disease with good tumor-related prognosis and long-life expectancy.

The findings of the current study, aiming to summarize the surgical anatomy and objective clinical data on TEAs through a systematic review of the current literature, have to be seen in light of some limitations. Although it shows the anatomical approaches in detail with dissections, it does not include a quantitative analysis and comparison of each approach. The literature contains clinical studies with heterogenity both in terms of pathologies and approaches as well as patient numbers due to the relatively new growing nature of these approaches which makes the generalization of the findings difficult.

## Conclusion

This study aimed to display each method of transorbital endoscopic surgery with anatomical dissections and to condense the data regarding this subject by making a systematic review of the current literature. It would be precise to comment that TEAs are important bricks in the wall of the endoscopic approaches to the SB. Data clustered so far indicate that these approaches provide important advantages reaching different pathologies and target areas through the SB. These versatile approaches allow the surgeon to avoid extra soft tissue dissection and provide a relatively short and direct corridor. They are not proposed to replace the transnasal or external approaches but are useful and important complementaries that should be in the armamentarium of a SB team.

## Supplementary Information

ESM 1 (DOCX 25 kb)

## Data Availability

Not applicable.

## References

[1] Almeida JP, Ruiz-Trevino AS, Shetty SR, Omay SB, Anand VK, Schwartz TH (2017). Transorbital endoscopic approach for exposure of the sylvian fissure, middle cerebral artery and crural cistern: an anatomical study. Acta Neurochir.

[2] Alqahtani A, Padoan G, Segnini G, Lepera D, Fortunato S, Dallan I, Pistochini A, Abdulrahman S, Abbate V, Hirt B, Castelnuovo P (2015). Transorbital transnasal endoscopic combined approach to the anterior and middle skull base: a laboratory investigation. Acta Otorhinolaryngol Ital.

[3] Balakrishnan K, Moe KS (2011). Applications and outcomes of orbital and transorbital endoscopic surgery. Otolaryngol Head Neck Surg.

[4] Bly RA, Su D, Hannaford B, Ferreira M, Moe KS (2012). Computer modeled multiportal approaches to the skull base. J Neurol Surg B Skull Base.

[5] Bly RA, Ramakrishna R, Ferreira M, Moe KS (2014). Lateral transorbital neuroendoscopic approach to the lateral cavernous sinus. J Neurol Surg B Skull Base.

[6] Bly RA, Ramakrishna R, Ferreira M, Moe KS (2014). Lateral transorbital neuroendoscopic approach to the lateral cavernous sinus. Journal of Neurological Surgery Part B. Skull Base.

[7] Cavallo LM, Somma T, Solari D, Iannuzzo G, Frio F, Baiano C, Cappabianca P (2019). Endoscopic endonasal transsphenoidal surgery: history and evolution. World Neurosurg.

[8] Chen HI, Bohman LE, Emery L, Martinez-Lage M, Richardson AG, Davis KA, Pollard JR, Litt B, Gausas RE, Lucas TH (2015). Lateral transorbital endoscopic access to the hippocampus, amygdala, and entorhinal cortex: ınitial clinical experience. ORL J Otorhinolaryngol Relat Spec.

[9] Ciporen JN, Moe KS, Ramanathan D, Lopez S, Ledesma E, Rostomily R, Sekhar LN (2010). Multiportal endoscopic approaches to the central skull base: a cadaveric study. World Neurosurg.

[10] Ciporen J, Lucke-Wold B, Dogan A, Cetas JS, Cameron WE (2016). Dual endoscopic endonasal transsphenoidal and precaruncular transorbital approaches for clipping of the cavernous carotid artery: a cadaveric simulation. J Neurol Surg B Skull Base.

[11] Ciporen JN, Lucke-Wold B, Dogan A, Cetas J, Cameron W (2017). Endoscopic endonasal transclival approach versus dual transorbital port technique for clip application to the posterior circulation: a cadaveric anatomical and cerebral circulation simulation study. J Neurol Surg B Skull Base.

[12] Dallan I, Castelnuovo P, Locatelli D, Turri-Zanoni M, AlQahtani A, Battaglia P, Hirt B, Sellari-Franceschini S (2015). Multiportal combined transorbital transnasal endoscopic approach for the management of selected skull base lesions: preliminary experience. World Neurosurg.

[13] Dallan I, Locatelli D, Turri-Zanoni M, Battaglia P, Lepera D, Galante N, Sellari-Franceschini S, Castelnuovo P (2015). Transorbital endoscopic assisted resection of a superior orbital fissure cavernous haemangioma: a technical case report. Eur Arch Otorhinolaryngol.

[14] Dallan I, Di Somma A, Prats-Galino A, Solari D, Alobid I, Turri-Zanoni M, Fiacchini G, Castelnuovo P, Catapano G, de Notaris M (2017). Endoscopic transorbital route to the cavernous sinus through the meningo-orbital band: a descriptive anatomical study. J Neurosurg.

[15] Dallan I, Sellari-Franceschini S, Turri-Zanoni M, de Notaris M, Fiacchini G, Fiorini FR, Battaglia P, Locatelli D, Castelnuovo P (2018). Endoscopic transorbital superior eyelid approach for the management of selected spheno-orbital meningiomas: preliminary experience. Oper Neurosurg (Hagerstown).

[16] De Rosa A, Pineda J, Cavallo LM, Di Somma A, Romano A, Topczewski TE, Somma T, Solari D, Ensenat J, Cappabianca P, Prats-Galino A (2019). Endoscopic endo- and extra-orbital corridors for spheno-orbital region: anatomic study with illustrative case. Acta Neurochir.

[17] Di Ieva A (2016). Lee JM.

[18] Di Somma A, Cavallo LM, de Notaris M, Solari D, Topczewski TE, Bernal-Sprekelsen M, Ensenat J, Prats-Galino A, Cappabianca P (2017). Endoscopic endonasal medial-to-lateral and transorbital lateral-to-medial optic nerve decompression: an anatomical study with surgical implications. J Neurosurg.

[19] Di Somma A, Andaluz N, Cavallo LM, de Notaris M, Dallan I, Solari D, Zimmer LA, Keller JT, Zuccarello M, Prats-Galino A, Cappabianca P (2018). Endoscopic transorbital superior eyelid approach: anatomical study from a neurosurgical perspective. J Neurosurg.

[20] Di Somma A, Andaluz N, Cavallo LM, Keller JT, Solari D, Zimmer LA, de Notaris M, Zuccarello M, Cappabianca P (2018). Supraorbital vs endo-orbital routes to the lateral skull base: a quantitative and qualitative anatomic study. Oper Neurosurg (Hagerstown).

[21] Di Somma A, Andaluz N, Cavallo LM, Topczewski TE, Frio F, Gerardi RM, Pineda J, Solari D, Ensenat J, Prats-Galino A, Cappabianca P (2018). Endoscopic transorbital route to the petrous apex: a feasibility anatomic study. Acta Neurochir.

[22] Di Somma A, Langdon C, de Notaris M, Reyes L, Ortiz-Perez S, Alobid I, Ensenat J (2020) Combined and simultaneous endoscopic endonasal and transorbital surgery for a Meckel's cave schwannoma: technical nuances of a mini-invasive, multiportal approach. J Neurosurg:1–10. 10.3171/2020.4.JNS20707 10.3171/2020.4.JNS20707 32650309

[23] Dindo D, Demartines N, Clavien PA (2004). Classification of surgical complications: a new proposal with evaluation in a cohort of 6336 patients and results of a survey. Ann Surg.

[24] Duz B, Secer HI, Gonul E (2009). Endoscopic approaches to the orbit: a cadaveric study. Minim Invasive Neurosurg.

[25] Ferrari M, Schreiber A, Mattavelli D, Belotti F, Rampinelli V, Lancini D, Doglietto F, Fontanella MM, Tschabitscher M, Rodella LF, Nicolai P (2016). The ınferolateral transorbital endoscopic approach: a preclinical anatomic study. World Neurosurg.

[26] Gassner HG, Schwan F, Schebesch KM (2015). Minimally invasive surgery of the anterior skull base: transorbital approaches. GMS Curr Top Otorhinolaryngol Head Neck Surg 14:Doc03. doi:10.3205/cto000118.

[27] Gerges MM, Godil SS, Younus I, Rezk M, Schwartz TH (2019). Endoscopic transorbital approach to the infratemporal fossa and parapharyngeal space: a cadaveric study. J Neurosurg.

[28] Golbin DA, Lasunin NV, Cherekaev VA, Grigorieva NN, Serova NK, Mindlin SN, Kutin MA, Imaev AA (2019). Biopsy and resection of skull base tumors using transorbital endoscopic approaches: primary results. Zh Vopr Neirokhir Im N N Burdenko.

[29] Jeon C, Hong CK, Woo KI, Hong SD, Nam DH, Lee JI, Choi JW, Seol HJ, Kong DS (2018) Endoscopic transorbital surgery for Meckel's cave and middle cranial fossa tumors: surgical technique and early results. J Neurosurg:1–10. 10.3171/2018.6.JNS181099 10.3171/2018.6.JNS181099 30544350

[30] Kasemsiri P, Carrau RL, Ditzel Filho LF, Prevedello DM, Otto BA, Old M, de Lara D, Kassam AB (2014). Advantages and limitations of endoscopic endonasal approaches to the skull base. World Neurosurg.

[31] Kong DS, Young SM, Hong CK, Kim YD, Hong SD, Choi JW, Seol HJ, Lee JI, Shin HJ, Nam DH, Woo KI (2018). Clinical and ophthalmological outcome of endoscopic transorbital surgery for cranioorbital tumors. J Neurosurg.

[32] Koppe M, Gleizal A, Orset E, Bachelet JT, Jouanneau E, Rougeot A (2013). Superior eyelid crease approach for transobital neuroendoscopic surgery of the anterior cranial fossa. J Craniofac Surg.

[33] Laleva L, Spiriev T, Dallan I, Prats-Galino A, Catapano G, Nakov V, de Notaris M (2019). Pure endoscopic lateral orbitotomy approach to the cavernous sinus, posterior, and ınfratemporal fossae: anatomic study. J Neurol Surg B Skull Base.

[34] Lee MH, Hong SD, Woo KI, Kim YD, Choi JW, Seol HJ, Lee JI, Shin HJ, Nam DH, Kong DS (2019). Endoscopic endonasal versus transorbital surgery for middle cranial fossa tumors: comparison of clinical outcomes based on surgical corridors. World Neurosurg.

[35] Li L, London NR, Chen X, Prevedello DM, Carrau RL (2020). Expanded exposure and detailed anatomic analysis of the superior orbital fissure: implications for endonasal and transorbital approaches. Head Neck.

[36] Lim JH, Sardesai MG, Ferreira M, Moe KS (2012). Transorbital neuroendoscopic management of sinogenic complications involving the frontal sinus, orbit, and anterior cranial fossa. J Neurol Surg B Skull Base.

[37] Lin BJ, Hong KT, Chung TT, Liu WH, Hueng DY, Chen YH, Ju DT, Ma HI, Liu MY, Hung HC, Tang CT (2019). Endoscopic transorbital transtentorial approach to middle incisural space: preclinical cadaveric study. Acta Neurochir.

[38] Lin BJ, Ju DT, Hsu TH, Chung TT, Liu WH, Hueng DY, Chen YH, Hsia CC, Ma HI, Liu MY, Hung HC, Tang CT (2019). Endoscopic transorbital approach to anterolateral skull base through inferior orbital fissure: a cadaveric study. Acta Neurochir.

[39] Locatelli D, Pozzi F, Turri-Zanoni M, Battaglia P, Santi L, Dallan I, Castelnuovo P (2016). Transorbital endoscopic approaches to the skull base: current concepts and future perspectives. J Neurosurg Sci.

[40] Lubbe D, Mustak H, Taylor A, Fagan J (2017) Minimally invasive endo-orbital approach to sphenoid wing meningiomas improves visual outcomes—our experience with the first seven cases. Clin Otolaryngol 42:876-880. doi:10.1111/coa.12722 10.1111/coa.12722 27529465

[41] Lubbe DE, Douglas-Jones P, Wasl H, Mustak H, Semple PL (2020). Contralateral precaruncular approach to the lateral sphenoid sinus-a case report detailing a new, multiportal approach to lesions, and defects in the lateral aspect of well-pneumatized sphenoid sinuses. Ear Nose Throat J.

[42] Lund VJ, Stammberger H, Nicolai P, Castelnuovo P, Beal T, Beham A, Bernal-Sprekelsen M, Braun H, Cappabianca P, Carrau R, Cavallo L, Clarici G, Draf W, Esposito F, Fernandez-Miranda J, Fokkens W, Gardner P, Gellner V, Hellquist H, Hermann P, Hosemann W, Howard D, Jones N, Jorissen M, Kassam A, Kelly D, Kurschel-Lackner S, Leong S, McLaughlin N, Maroldi R, Minovi A, Mokry M, Onerci M, Ong YK, Prevedello D, Saleh H, Sehti DS, Simmen D, Snyderman C, Solares A, Spittle M, Stamm A, Tomazic P, Trimarchi M, Unger F, Wormald PJ, Zanation A, European Rhinologic Society Advisory Board on Endoscopic Techniques in the Management of Nose PS, Skull Base T (2010). European position paper on endoscopic management of tumours of the nose, paranasal sinuses and skull base. Rhinol Suppl.

[43] Lyson T, Sieskiewicz A, Rogowski M, Mariak Z (2014). Endoscopic lateral orbitotomy. Acta Neurochir.

[44] Matsuo S, Komune N, Iihara K, Rhoton AL (2016). Translateral orbital wall approach to the orbit and cavernous sinus: anatomic study. Oper Neurosurg (Hagerstown).

[45] Meccariello G, Deganello A, Choussy O, Gallo O, Vitali D, De Raucourt D, Georgalas C (2016). Endoscopic nasal versus open approach for the management of sinonasal adenocarcinoma: a pooled-analysis of 1826 patients. Head Neck.

[46] Moe KS (2014). Transorbital endoscopic approaches to the anterior cranial fossa. In: Gardner P. Synerderman Carl H (ed) Master techniques in otolaryngology-head and neck surgery: skull base surgery 1st Edition.

[47] Moe KS, Bergeron CM, Ellenbogen RG (2010). Transorbital neuroendoscopic surgery. Neurosurgery.

[48] Moe KS, Kim LJ, Bergeron CM (2011). Transorbital endoscopic repair of cerebrospinal fluid leaks. Laryngoscope.

[49] Nicolai P, Ferrari M, Maroldi R (2019) Endoscopic transnasal anatomy of the skull base and adjacent areas: a lab dissection and radiological atlas. Thieme

[50] Noiphithak R, Yanez-Siller JC, Revuelta Barbero JM, Otto BA, Carrau RL, Prevedello DM (2018). Quantitative analysis of the surgical exposure and surgical freedom between transcranial and transorbital endoscopic anterior petrosectomies to the posterior fossa. J Neurosurg.

[51] Noiphithak R, Yanez-Siller JC, Revuelta Barbero JM, Cho RI, Otto BA, Carrau RL, Prevedello DM (2019). Comparative analysis of the exposure and surgical freedom of the endoscopic extended minipterional craniotomy and the transorbital endoscopic approach to the anterior and middle cranial fossae. Oper Neurosurg (Hagerstown).

[52] Noiphithak R, Yanez-Siller JC, Revuelta Barbero JM, Otto BA, Carrau RL, Prevedello DM (2019). Comparative analysis between lateral orbital rim preservation and osteotomy for transorbital endoscopic approaches to the cavernous sinus: an anatomic study. Oper Neurosurg (Hagerstown).

[53] Park HH, Yoo J, Yun IS, Hong CK (2020) Comparative analysis of endoscopic transorbital approach and extended mini-pterional approach for sphenoid wing meningiomas with osseous ınvolvement: preliminary surgical results. World Neurosurg. 10.1016/j.wneu.2020.01.115 10.1016/j.wneu.2020.01.115 32001400

[54] Patel CR, Fernandez-Miranda JC, Wang WH, Wang EW (2016). Skull base anatomy. Otolaryngol Clin N Am.

[55] Prabhakaran VC, Selva D (2008). Orbital endoscopic surgery. Indian J Ophthalmol.

[56] Priddy BH, Nunes CF, Beer-Furlan A, Carrau R, Dallan I, Prevedello DM (2017). A side door to Meckel's cave: anatomic feasibility study for the lateral transorbital approach. Oper Neurosurg (Hagerstown).

[57] Ramakrishna R, Kim LJ, Bly RA, Moe K, Ferreira M (2016). Transorbital neuroendoscopic surgery for the treatment of skull base lesions. J Clin Neurosci.

[58] Raza SM, Quinones-Hinojosa A, Lim M, Boahene KD (2013). The transconjunctival transorbital approach: a keyhole approach to the midline anterior skull base. World Neurosurg.

[59] Saraceno G, Agosti E, Qiu J, Buffoli B, Ferrari M, Raffetti E, Belotti F, Ravanelli M, Mattavelli D, Schreiber A, Hirtler L, Rodella LF, Maroldi R, Nicolai P, Gentili F, Kucharczyk W, Fontanella MM, Doglietto F (2020). Quantitative anatomical comparison of anterior, anterolateral and lateral, microsurgical and endoscopic approaches to the middle cranial fossa. World Neurosurg.

[60] Snyderman C, Gardner P (2014) Master techniques in otolaryngology-head and neck surgery: skull base surgery. Lippincott Williams & Wilkins

[61] Topczewski TE, Di Somma A, Pineda J, Ferres A, Torales J, Reyes L, Morillas R, Solari D, Cavallo LM, Cappabianca P, Ensenat J, Prats-Galino A (2020). Endoscopic endonasal and transorbital routes to the petrous apex: anatomic comparative study of two pathways. Acta Neurochir.

[62] Verillaud B, Bresson D, Sauvaget E, Mandonnet E, Georges B, Kania R, Herman P (2012). Endoscopic endonasal skull base surgery. Eur Ann Otorhinolaryngol Head Neck Dis.

[63] Zwagerman NT, Zenonos G, Lieber S, Wang WH, Wang EW, Fernandez-Miranda JC, Snyderman CH, Gardner PA (2016). Endoscopic transnasal skull base surgery: pushing the boundaries. J Neuro-Oncol.

